# A role for pH dynamics regulating transcription factor DNA-binding selectivity

**DOI:** 10.1093/nar/gkaf474

**Published:** 2025-06-04

**Authors:** Kyle P Kisor, Diego Garrido Ruiz, Matthew P Jacobson, Diane L Barber

**Affiliations:** Department of Cell and Tissue Biology, University of California San Francisco, San Francisco, CA 94143, United States; Department of Pharmaceutical Chemistry, University of California San Francisco, San Francisco, CA 94143, United States; Department of Pharmaceutical Chemistry, University of California San Francisco, San Francisco, CA 94143, United States; Department of Cell and Tissue Biology, University of California San Francisco, San Francisco, CA 94143, United States

## Abstract

Intracellular pH (pHi) dynamics regulates diverse cell processes such as proliferation, dysplasia, and differentiation, often mediated by the protonation state of a functionally critical histidine residue in endogenous pH sensing proteins. How pHi dynamics can directly regulate gene expression or whether transcription factors can function as pH sensors has received limited attention. We tested the prediction that transcription factors with a histidine in their DNA-binding domain (DBD) that forms hydrogen bonds with nucleotides can have pH-regulated activity, which is relevant to more than 85 transcription factors in distinct families, including FOX, KLF, SOX, and MITF/Myc. Focusing on FOX family transcription factors, we use unbiased SELEX-seq to identify pH-dependent DNA-binding motif preferences and confirm pH-regulated binding affinities for FOXC2, FOXM1, and FOXN1 to a canonical FkhP DNA motif that are greater at pH 7.0 compared with pH 7.5 and for FOXN1 to a preferred FHL motif at higher pHi in cells. For FOXC2, we also find that greater activity for an FkhP motif at lower pH is dependent on a conserved histidine (His122) in the DBD. ChIP-seq and RNA-seq with FOXC2 also reveal pH-dependent differences in enriched promoter motifs. Our findings identify pH-regulated transcription factor-DNA binding selectivity with relevance to how pHi dynamics can regulate gene expression for myriad cell behaviours.

## Graphical Abstract

**Graphical Abstract:**
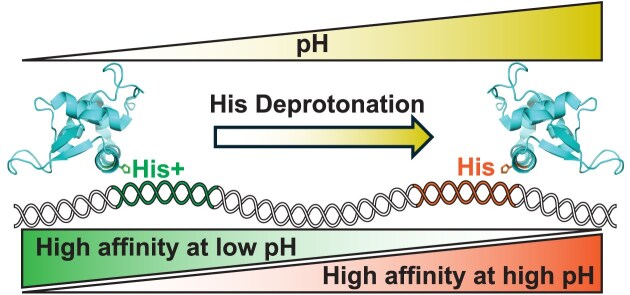

## Introduction

Intracellular pH (pHi) dynamics is increasingly recognized as a critical regulatory signal for many cell behaviours, including cell proliferation, migration, and differentiation. Although changes in pHi were previously viewed as a mechanism to maintain homeostasis, it is now known that changes in pHi occur and are necessary for normal cell processes such as cell cycle progression for proliferation [1–3], actin filament and cell–substrate adhesion remodeling for directed cell migration [4–9], and stem cell differentiation and lineage specification [10–12]. Moreover, it is now established that pHi dynamics is dysregulated in human diseases, including cancer [13–15], diabetes [16, 17], and neurodegeneration [18, 19]. The molecular mechanisms by which pHi dynamics regulates normal and pathological cell behaviours is often mediated by protein electrostatics and the protonation state of key residues in endogenous pH sensitive proteins termed “pH sensors” [20, 21].

Of all amino acids that undergo protonation state changes in solution, histidine side chains have the closest p*K*a [6.5] to cellular pH. However, the p*K*a of histidine, aspartic acid, glutamic acid, and buried lysine can be upshifted or downshifted depending on the protein landscape and electrostatic environment to enable titration within the cellular pH range of 7.0–7.8 [6, 22, 23]. Changes in the protonation state of key amino acids in response to small changes in pH (0.2–0.4) can significantly alter protein structure and function, including activity [7, 24–26] and ligand binding [5, 6, 27–29] as well as stability [30–32] and aggregation [33, 34]. While many cell processes regulated by pHi dynamics include changes in gene expression [3, 12, 35], and although nuclear and cytosolic pH are similar [36], how pHi can regulate transcription factor target gene selectivity remains understudied and unclear.

Our current understanding of how transcription factors achieve DNA-binding specificity includes distinct regulatory mechanisms, such as co-factor association, post-translational modifications such as phosphorylation, and cell-specific expression [37–39] as well as DNA architecture [40–43]. Here, we tested the prediction that DNA-binding specificity of transcription factors with a histidine in the DNA-binding domain (DBD) that interacts directly with nucleotides might be regulated by pHi dynamics. Significantly, this prediction could be relevant to >85 transcription factors in distinct families, including FOX, KLF, SOX, and MITF/Myc, which in all available structures in complex with DNA contain a histidine in the DBD that directly forms a hydrogen bond with nucleotides.

We confirmed our prediction by showing pH-regulated DNA binding of three FOX family transcription factors: FOXC2, FOXM1, and FOXN1. For FOXC2 and FOXN1, we find pHi regulated activity in cells, and for FOXC2, we show the critical importance of a conserved histidine for pH-sensitive DNA binding and gene expression. Additionally, we used unbiased approaches of SELEX-seq, ChIP-seq, and RNA-seq to identify different DNA motif binding profiles at lower pH (7.0–7.4) compared with higher pH (7.7–7.8) and a preference for binding thymine at lower pH. Our findings fill gaps in our current understanding not only for how pHi dynamics can tune selectivity for target genes and regulate gene expression but also for how transcription factors with highly similar DNA-binding domains can regulate diverse genes and functions reiteratively for developmental programs that include changes in pHi [11, 44].

## Materials and methods

### Amino acid sequence and structural alignment

For sequence alignment of FOX, KLF, SOX, and MITF/MYC/MAX family proteins, FASTA sequences were downloaded from UniProt and uploaded to Jalview software. For each family, sequences were aligned using ClustalO default settings. Amino acids were arbitrarily colored with red used to highlight the conserved DNA-binding histidine in each family. For structural alignment, available FOX crystal structures in complex with DNA were downloaded from the Protein Data Bank (PDB), which included FOXA2 (5 × 07), FOXC2 (6AKO), FOXG1 (7CBY), FOXH1 (7YZ7), FOXK2 (2C6Y), FOXL2 (7VOU), FOXM1 (3G73), FOXN1 (6EL8), FOXN3 (6NCE), FOXO1a (3CO6), FOXO3 (2UZK), FOXO4 (3L2C), FOXP2 (2AS5), and FOXP3 (7TDW). Structures were aligned using FOXC2 (green) as a reference for other FOX structures shown in complex with DNA (grey) by using PyMOL software.

### Recombinant protein cloning, expression, and purification

The GST-fusion protein plasmid pGEX-6P-2 was digested with BamHI and EcoRI enzymes and the DBD of FOXC2 (amino acids 72–172), FOXM1 (amino acids 222–360), and FOXN1 (amino acids 270–366) were amplified by polymerase chain reaction (PCR) with BamHI and EcoRI cloning site overhangs. FOX templates were obtained from Addgene [45] and FOX-DBD DNA sequences were ligated and cloned into the pGEX-6P2 using Gibson Assembly Master Mix (NEB: E2611L). Point mutants were generated with the QuikChange Lightning site-directed mutagenesis kit (Agilent: 210515), according to the manufacturer’s protocol. Each construct was transformed into and expressed in BL21-DE3 *Escherichia coli* competent cells using heat shock (Thermo EC0114). For expression, cells were grown in 1L of Luria broth with ampicillin (100 μg/ml; 37°C with shaking at 225 rpm) until cells reached log-phase growth at OD_600_ = ∼0.6. Expression was induced with 1 mM isopropyl-β-d-1-thiogalatapyranoside for 6 h at 37°C with shaking at 225 rpm. Cells were pelleted (7000 × *g*; 15 min at 4°C) and either frozen at −80°C or used directly for protein purification.

Bacterial cell pellets were resuspended in 50 ml of lysis buffer (50 mM Tris–HCl pH 8, 1 mM dithiothreitol [DTT], 5% glycerol, protease inhibitor cocktail [Roche 1183615300]). Cells in pellets were lysed by sonication on ice with 10 s pulses of maximum setting followed by 1 min cooling period, repeated 12 times. The supernatant was clarified by centrifugation (12 000 × g; 30 min at 4°C) and mixed 1:1 with wash buffer (50 mM Tris, 150 mM NaCl, pH 8.0) from a GST purification kit (Pierce: 16105). 12.5 ml of lysate was loaded on pre-equilibrated 3 ml of glutathione agarose spin columns, incubated end over end for 30 min at 4°C, and repeated until all lysate was used. The flowthrough was collected by centrifugation (700 × *g*; 2 min at 4°C) and columns were washed with 6 ml of wash buffer three times. GST-FOX DBDs were eluted with 3 ml of wash buffer containing 10 mM reduced glutathione three times. Each fraction was collected, separated on a 10% sodium dodecyl sulfate–polyacrylamide gel electrophoresis (SDS–PAGE) gel, and Coomasie stained to determine molecular size and purity. Eluate fractions were pooled, divided in half, concentrated, and exchanged in two separate anisotropy buffers (20 mM HEPES, 140 mM KCl, 0.05 mM TCEP–HCl, pH 7 or 7.5) using Amicon Ultra-15 Filters with a 10 kDa molecular weight cutoff (MilliporeSigma: UFC901008). The protein concentration was determined by using a NanoDrop spectrophotometer (Thermo: ND-1000), and samples were aliquoted, flash frozen in liquid nitrogen, and stored at −80°C.

### SELEX-seq for identifying pH-dependent binding sequences

For SELEX-seq, a previously reported protocol [46] was used with modifications. We designed a library with a 16 base pair randomized region flanked by PCR sites GTTCAGAGTTCTACAGTCCGACGATCTGGNNNNNNNNNNNNNNNNTCGTATGCCGTCTTCTGCTTG. For each round of selective enrichment, final concentrations of 0.25 μM DNA library with 2.5 μM GST-FOXC2 were incubated for 30 min at RT in binding buffer (20 mM HEPES, 140 mM KCl, and 0.05 mM TCEP–HCl) at either pH 7 or 7.8. Next, 30 μl of 50% pre-equilibrated glutathione Sepharose beads (Cytiva: 17075601) were added, and samples were incubated end over end for 30 min at RT after which DNA–FOXC2–bead complexes were pelleted by centrifugation at 5000 rpm for 4 min. DNA–FOXC2–bead complexes were washed twice with 300 μl of binding buffer and resuspended in 100 μl of binding buffer prior to heat dissociation for 5 min at 95°C. Eluate DNA was clarified by centrifugation (10 000 rpm for 5 min) and PCR amplified with forward (SELEX_F_) and reverse (SELEX_R_) primers complementary to nucleotides flanking the randomized region (Table 1). PCR products were cleaned using the MinElute PCR purification kit (Qiagen 28004), and the selection process was repeated four times with PCR products from prior rounds used as the starting library. 5′ and 3′ adapter overhangs were added by PCR (Table 2) to the initial starting library (*R*_0_) with all four rounds of selection (*R*_1–4_) used to prepare for addition of barcoded indexes. Indexes were added by PCR (Table 2) using the Nextera IDT UD Set D (Illumina 20027213) for multiplex sequencing. Sequencing was performed at the University of California, San Francisco Genomics CoLab using a Miniseq high output 150 cycles kit for paired end reads (Illumina FC-420-1002). Samples were demultiplexed and analysis performed using the University of California, Davis Bioinformatics Core according to the protocol outlined in [46] using their published ‘R’ “SELEX” package which is publicly available at bioconductor.org/packages/release/bioc/html/SELEX.html.

**Table 1.** Primers used in SELEX-seq library

Step	Primer	Sequence 5′>3′
Library amplification	SELEX_F_	GTTCAGAGTTCTACAG
		TCCGACGATCTGG
	SELEX_R_	CGAAGTCAAGCAGAAG
		ACGGCATACGA
Adapter overhangs	Overhang_F_	TCGTCGGCAGCGTCA
		GATGTGTATAAGAGAC
		AGGTTCAGAGTTCTAC
		AGTCCGACGATC
	Overhang_R_	GTCTCGTGGGCTCGG
		AGATGTGTATAAGAGA
		CAGCGAAGTCAAGCAG
		AAGACGGCATAC
Barcoded indexes	Barcode_F_	AATGATACGGCGACCA
		CCGAGATCTACACNNN
		NNNNNTCGTCGGCAGC
		GTC
	Barcode_R_	CAAGCAGAAGACGGCA
		TACGAGATNNNNNNNN
		GTCTCGTGGGCTCGG

**Table 2.** Indexes from Nextera IDT UD Set D used for each sample

Sample	Index ID	Index 5′	Index 3′
R_0_ Initial Library	UDP0337	TGTAAGGTGG	AAGGCCTTGG
R_1_ FOXC2 pH 7.0	UDP0338	CAACTGCAAC	TGAACGCAAC
R_1_ FOXC2 pH 7.8	UDP0339	ACATGAGTGA	CCGCTTAGCT
R_2_ FOXC2 pH 7.0	UDP0340	GCAACCAGTC	CACCGAGGAA
R_2_ FOXC2 pH 7.8	UDP0341	GAGCGACGAT	CGTATAATCA
R_3_ FOXC2 pH 7.0	UDP0342	CGAACGCACC	ATGACAGAAC
R_3_ FOXC2 pH 7.8	UDP0343	TCTTACGCCG	ATTCATTGCA
R_4_ FOXC2 pH 7.0	UDP0344	AGCTGATGTC	TCATGTCCTG
R_4_ FOXC2 pH 7.8	UDP0304	CCGCTCCGTT	TACGGCGAAG

### Constant pH molecular dynamics

Constant pH molecular dynamics (CpHMD) simulations were performed to estimate protonation state distributions for defined titratable histidine residues in FOXC2 and FOXM1 in thermodynamic equilibrium, both as protein–DNA complexes and as solvated monomers [47] . The p*K*as of histidine residues were estimated to inform how DNA–protein interaction is modulated as a function of changes in pH through proton uptake and release [48]. All CpHMD simulations were run for 100 ns at pH 7.0, with protonation state change attempts every 100 fs. Predicted p*K*a of titratable residues were estimated using DelPhiPKa [49, 50].

### GST-FOX DBD anisotropy

GST-FOX DBD protein aliquots were thawed at room temperature and diluted to a final volume of 120 μl in anisotropy buffer pH 7 and 7.5 to the highest concentration of each protein (FOXC2: 50 μM, FOXM1: 21 μM, FOXN1: 50 μM, FOXC2-H122K: 9 μM, FOXC2-H122N: 97 μM, FOXM1-H287K: 21 μM) needed to reach binding saturation, which was empirically determined. Protein was serially diluted ten times to a volume of 60 μl, and 10 μl of 6′Carboxyfluorescein (FAM)-labeled duplex FkhP (ccATAAACAac) (IDT) was added to each protein dilution and one blank for a final concentration of 7.5 nM DNA and 70 μl reaction volume. PCR strip tubes were capped and incubated at RT in the dark for 30 min. Following incubation, 20 μl of each dilution and blank was loaded in triplicate to a 384-well black plate (Greiner: 784076) using a multichannel pipette. Fluorescence anisotropy measurements were made using a SpectraMax M5 plate reader (Molecular Devices). Sigmoidal curve fits were generated using GraphPad Prism and association constants determined using Mathematica software to solve for *K*_D_ in equation ${A_{{\rm obs}}} = {A_0} + (\Delta A*T)/({K_D} + T)$ where *A*_obs_ is observed anisotropy, *A*_0_ is anisotropy of initial unbound probe, Δ*A* is difference in anisotropy between unbound and fully bound populations, and *T* is concentration of titrant protein. *K*_A_ was determined as (1/*K*_D_). Data are expressed as averages of at least three independent measurements from two to three separate protein preparations, and error bars are ± s.e.m. Binding affinities of FOX wild-type (WT) proteins were analysed by two-tailed unpaired Student’s *t*-test and with a significance level of *P* < 0.05. Comparison of WT FOXC2 and FOXM1 to His mutants was analysed by Tukey–Kramer HSD with a significance level of *P* < 0.05.

For GST-FOXC2 DBD pH titration anisotropy buffer was prepared in increments of 0.2 pH units from pH 6.8 to 8.2, and 7.5 nM final concentration of labeled FkhP oligo was added. Either 2.3 μM final concentration GST-FOXC2 or buffer alone was added to a final reaction volume of 70 μl. Reactions were incubated and measured as described above. Data are represented as average anisotropy values of [(FOXC2 + DNA) – (DNA alone)] set relative to maximal binding at pH 6.8 ± s.e.m.

### Cell culture and reporter assays

MDA-MB-436 cells obtained from ATCC were maintained at 37°C and 5% CO_2_ in RPMI 1640 (Gibco:11875085) supplemented with penicillin/streptomycin (100U/ml each) and 10% FBS, and were commercially authenticated (IDEXX BioAnalytics). To lower pHi, MDA-MB-436 cells were treated with 5-(*N*-ethyl-*N*-isopropyl)-amiloride (EIPA; 10 μM for 24 h) or silenced for NHE1 by using CRISPR–Cas9 gene editing. For CRISPR–Cas9 knock out of NHE1 (NHE1-KO), we used a protocol we recently described with guide RNA (5′- CACCGGTTTGCCAACTACGAACACG-3′) and reverse (5′-AAACCGTGTTCGTAGTTGGCAAACC-3′) targeting the first exon of the NHE1 gene SLC9A1, including fluorescence activated sorting to select for cells transiently expressing Cas9-GFP [11]. Loss of NHE1 activity was confirmed as described below in pHi determinations.

For luciferase assays, 3.5 × 10^5^ MDA-MB-436 cells were plated in six-well plates, grown overnight to 80% confluency, and then transfected with a total of 1 μg DNA using Lipofectamine 3000 (Invitrogen L3000001) according to the manufacturer’s protocol. Cells received either 500 ng 6x-FkhP (6x-DBE) obtained from Addgene [45] or 500 ng 6x-FkhP with 500 ng pCS2 Flag-FOXC2 WT from Addgene [45] or mutant FOXC2 generated as described above. Each transfection also included control pRL-TK renillla plasmid obtained from the L. Selleri Lab (University of California San Francisco) at a ratio of 1:10 of reporter plasmid (50 ng). At 8 h after transfection, cells were washed once with PBS and growth medium added in the absence or presence of 10 μM EIPA. Cells were then maintained until collected for Dual-Luciferase assays (Promega: E2920). In brief, cells were washed with PBS and lysed in 500 μl of Dual-Glo luciferase buffer with shaking on a nutator for 10 min at 4°C. Lysates were collected in microfuge tubes and clarified by centrifugation for 5 min at 13 000 rpm at RT. From supernatants, 100 μl was loaded in quadruplicate in separate wells in a 96-well opaque white plate (Costar: 3917). Luciferase signal was read on a SpectraMax M5 plate reader, and Dual-Glo Stop & Glo Buffer was then added to quench the luciferase signal and activate renilla for 10 min. The renilla signal was read and the Luciferase/Renilla ratio was normalized to control with 6x-FkhP + WT FOXC2. Data were analysed by Tukey–Kramer HSD with a significance level of *P* < 0.05.

For fluorescence dual reporter assays, we designed a reporter with a 6x repeat of FHL driving expression of mKate and a 6x repeat of FkhP driving expression of eGFP. Cells were plated on 35mm MatTek dishes (MatTek Corporation; P35G-1.5-10-C) and transfected as described for luciferase assays but with either 500 ng dual reporter or 500 ng dual reporter with 500 ng of pCS2 Flag-FOXN1. Fluorescence images for eGFP (*E*x: 488 nm) and mKate (*E*x: 561 nm) were obtained using a Plan Apo 40 0.95 NA objective on an inverted Nikon spinning disc microscope system (Nikon Eclipse TE2000 Perfect Focus System; Nikon Instruments; Nikon Instruments) equipped with a CoolSnap HQ2 cooled charge-coupled camera (Photometrics) and camera-triggered electronic shutters controlled with NIS-Elements Imaging Software (Nikon). For image analysis, we used NIS software to quantify fluorescence intensities and ratios for at least five separate images from three independent cell preparations. In brief, after background subtraction, the mean fluorescence intensities for entire images obtained at *E*x 488 and 561 nm were quantified and ratios determined. Data were analysed by Tukey–Kramer HSD.

### pHi determinations

For imaging cytosolic and nuclear pH, MDA-MB-436 cells plated on 35 mm glass bottom MatTek dishes for 48 h were washed 2× and incubated for 15 min in pHi buffer (110 mM NaCl, 5 mM KCl, 10 mM glucose, 25 mM NaHCO_3_, 1 mM KPO_4_, 1 mM MgSO_4_, 2 mM CaCl_2_, pH 7.4) containing 10 μM of the dual emission pH-sensitive dye 5-(and-6)-carboxylic acid, acetoxymethyl ester (SNARF) as previously described [51]. For measurements with EIPA, a final concentration of 10 μM was included in cell medium 24 h before measurements and in all wash and dye-loading buffers. After dye loading and washing 2× in pHi buffer, ratiometric determinations at Ex 490 were made at pH-sensitive and -insensitive emissions of 580 and 640 nm, respectively. Dye ratios with imaging and as described below for cell populations were calibrated to pHi values by incubating cells at the end of each determination sequentially for 5 min each with a Na^+^-free, K^+^ buffer containing the ionophore nigericin at pH 7.5 and then at pH 6.6 to equilibrate intracellular and extracellular pH, as previously described [51, 52]. SNARF fluorescence was imaged by using a Nikon microscope system as described above for dual fluorescence reporter imaging.

For measuring pHi in cell populations, parental MDA-MB-436 cells and NHE1-KO MDA-MB-436 cells were plated at 25 000 cells/well in 24-well dishes for 48 h. After 24 h, EIPA (10 μM) was added where indicated and included in all buffers for pHi measurements. Cells were washed 2× and incubated for 15 min in pHi buffer containing 1 μM of the dual excitation pH-sensitive dye 2′,7′-bis-(2-carboxyethyl)-5-(and-6)-carboxyfluorescein, acetoxymethyl ester (BCECF) as previously described [52]. After dye loading and washing 2× in pHi buffer, ratiometric determinations were made at *E*m 535 nm and pH-sensitive and -insensitive excitations of 490 and 440 nm, respectively, using a SpectraMax M5 plate reader [47, 48]. To confirm loss of NHE1 activity with EIPA or with NHE1-KO, the rate of H + efflux was determined in a nominally HCO_3_-free HEPES buffer (145 mM NaCl, 5 mM KCl, 10 mM glucose, 25 mM HEPES, 1 mM MgSO_4_, 1 mM KPO_4_, 2 mM CaCl_2_, pH 7.4) by measuring the time-dependent pHi recovery from an NH_4_Cl-induced acid load as previously described [52].

### Immunoblotting

Immunoblots were performed using antibodies for anti-FLAG (Sigma A8592), anti-FOXC2 (Abcam ab5060), and anti-GAPDH (Cell Signaling Technologies 2118s). For immunoblots using luciferase assay lysates, 20 μl of remaining supernatant from Dual-Glo lysis reaction was prepared with 1× Laemmli loading buffer and loaded on a 10% tris–glycine gel (Bio-Rad 4560133). Samples from all other immunoblots were generated by full RIPA lysis, prepared in 1× Laemmli buffer, and loaded at a total of 20 μg of total protein per lane. Following electrophoresis and transfer to PVDF membranes (Millipore IPVH00010), membranes were blocked with 5% nonfat dry milk (NFDM) in TBST and incubated at RT for 1 h and probed with each antibody at 1:1000 overnight at 4C. For detection, blots were incubated for 1 h at RT with horseradish peroxidase-conjugated secondary antibodies; for anti-FOXC2 donkey anti-goat (Invitrogen PA1-28664), for GAPDH (Cell Signaling Technologies 7074s) both at 1:10 000 in NFDM. Anti-FLAG is horseradish peroxidase pre-conjugated and does not require secondary antibody. Blots were briefly incubated in horseradish peroxidase-chemiluminescent substrate (Thermo 34 096) and visualized on Bio-Rad ChemiDoc XRS+ .

### ChIP-seq sample processing and data analysis

MDA-MB-436 cells were maintained as described for luciferase assays and plated in duplicate at 1 × 10^7^ control or NHE1-KO cells per 100 mm plate and grown overnight to 80% confluency prior to transfection. Cells were transfected with 12.5 μg of WT Flag-FOXC2 and medium changed 8 h after transfection. Cells were incubated for 48 h after transfection and were fixed for 5 min with 1% formaldehyde at room temperature. Fixation was stopped with 117 mM glycine and washed with cold phosphate-buffered saline (PBS). Cells were lysed (50 mM Tris–HCl pH 8.0, 2 mM EDTA, 0.1% NP-40, 10% glycerol, 1 mM phenylmethylsulfonyl fluoride [PMSF]) and nuclei collected. Nuclei were then lysed with (50 mM Tris–HCl pH 8.0, 10 mM EDTA, 1% SDS, 1 mM PMSF) and lysates sheared by sonication with 30 s on/off for 5 min repeated eight times with a 2 min rest between each cycle. Effective shearing to between 200 and 1000 bp was confirmed by separating on agarose gels and chromatin was frozen on dry ice and stored at −80°C. For immunoprecipitation, thawed samples were diluted 1:10, precleared with Protein G Dynabeads (Thermo 10003D), and input sample collected. Samples were then incubated overnight with 10 μg anti-Flag antibodies (Sigma-F1804) overnight. Immunoprecipitations were collected using Protein G Dynabeads with extensive washing to minimize nonspecific immunoprecipitation [4× wash buffer 1 (0.1% SDS, 1% NP-40, 2 mM EDTA, 500 mM NaCl, 20 mM Tris–HCl pH 8.0, 2 mM PMSF), 3× wash buffer 2 (0.1% SDS, 1% NP-40, 2 mM EDTA, 0.5 M LiCl, 20 mM Tris–HCl pH 8.0, 2 mM PMSF), and 3× wash buffer 3 (1 mM EDTA, 10 mM Tris–HCl pH 8.0)]. Samples were eluted, de-crosslinked overnight, and digested with proteinase K (Thermo EO0491). All steps were performed at 4°C or on ice unless stated otherwise. DNA was then purified using MicroChIP DiaPure columns (Diagenode C03040001) and stored at −80°C. Samples were processed by Azenta Life Sciences Co. Ltd (USA) for DNA quality assessment, library construction, sequencing on an Illumina MiSeq, data acquisition, and peak calling. Data are from three paired biological replicates of each condition. Counts of unique and shared peaks were analysed using BioVenn python package [53]. Enriched *de novo* unique peaks from control wild-type cells (pHi 7.7) compared with NHE1-KO cells (pHi 7.4) were analysed for DNA motifs using the HOMER package findMotifsGenome.pI [54]. The most common DNA motif enriched at pHi 7.7 compared with pHi 7.4 were compared using the DiffLogo [55] R-package. Representative peak tracks differentially enriched between controls and NHE1-KO are shown at 5 kb scale using Integrative Genomics Viewer (IGV) [56] .

### Library preparation, RNA-seq, and analysis

MDA-MB-436 cells were maintained as described for luciferase assays and plated at 3.5 × 10^5^ control or NHE1-KO cells per well in 6 WP and grown overnight to 80% confluency prior to transfection. Cells were either untransfected or transfected with 1 μg of FOXC2-WT, -H122K, or -H122N with medium changed 8 h after transfection. Cells were incubated for 48 h post transfection and RNA was extracted with the RNeasy Mini kit (Qiagen, 74104) according to manufacturer’s instructions with sample concentrations assessed via NanoDrop. Samples were frozen at −80°C and sent to Novogene Co. Ltd (USA) for library RNA sample quality assessment, library construction, RNA-sequencing on an Illumina NovaSeq 6000, read mapping, and statistical analysis of differentially expressed genes (DEGs). Data are from three paired biological replicates of each condition. DEGs in each condition were analysed using InteractiVenn web tool [57]. Volcano plot analysis was performed with a custom Python script with a significance value cutoff of (qval < −log_10_ (0.05)). Select normalized gene fold change between controls and NHE1-KO cells for untransfected, FOXC2-WT, -H122K, or -H122N was generated with GraphPad Prism. Promoter elements from gene lists in the human hg19 genome at −2000 to + 100 base pairs from the transcription start site were analysed using the HOMER FindMotifs [54] script. The most common promoter elements enriched at pHi 7.7 versus pHi 7.4 were compared using the DiffLogo [55] R package with statistical analysis of position weight matrix difference performed with Kullback–Leibler divergence.

## Results

### Predicted pH-sensing by transcription factors with a conserved histidine in the DBD

**Figure 1.**
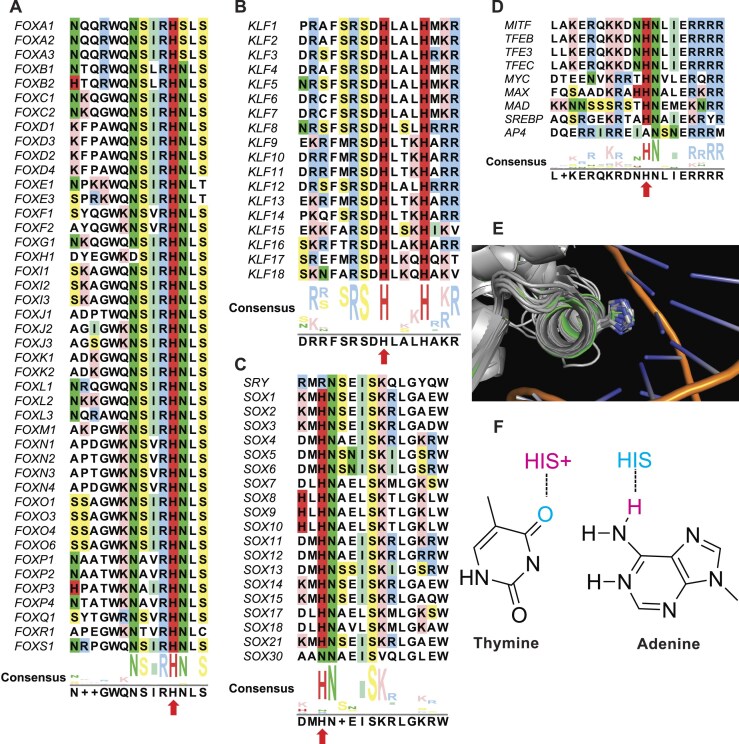
FOX family proteins with predicted pH-regulated DNA binding. (**A**–**D**) Sequence alignment of Helix H3 of the DBD of FOX (A), KLF (B), SOX (C), and MITF/MYC/MAX (D) family members with conserved histidine highlighted in red and denoted by red arrows. (**E**) Alignment of all available crystal structures of FOX proteins in complex with DNA highlighting spatial alignment the side chain of the conserved histidine. (**F**) Proposed model for pH-regulated transcription factor-DNA binding.

We previously described the design principles of several pH-sensors that are regulated by titration of a histidine within the cellular pH range of 7.0–7.8 [5–7, 26, 30, 58]. In asking whether some transcription factors might function as pH sensors we searched for conserved histidine residues important for DNA binding across transcription factor families. We first performed sequence alignments of major transcription factor family members expressed in humans. We find that all FOX family members contain a histidine in a highly conserved N(S/A)IRH motif within Helix 3 of the DBD (Fig. 1A). In all available crystal structures of FOX transcription factors in complex with DNA, the conserved histidine aligns in the major groove of DNA and forms a hydrogen bond with nucleotides. Further, we used a structural overlay to show that the position of the side chain of conserved histidine residues is spatially conserved (Fig. 1E). We also find that all members of the KLF transcription factor family, all members of the SOX transcription factor family except Sry and SOX30, and all members of the MITF/Myc/Max family except AP4 contain a conserved histidine in the DBD, which in available structures in complex with DNA forms a hydrogen bond with nucleotides (Fig. 1B–D and Supplementary Fig. S1A–C). Additionally, a hydrogen bond between a histidine in the DBD and DNA nucleotides is reported for the ETS transcription factor ETV6 [59], the STAT transcription factor STAT6 [60], and ARNT, the DNA-binding subunit of the HIF1 complex [61, 62] (Supplementary Fig. S1D–F). Hence, at least 85 transcription factors in diverse families contain a histidine in the DBD that in available structures forms a hydrogen bond with nucleotides.

Given the conservation of a histidine in the DBD of many transcription factors that forms a hydrogen bond with nucleotides, coupled with the well-established ability of histidine to titrate within the cellular pH range, we hypothesized that a pH-dependent titration of the conserved histidine might regulate DNA-binding specificity through hydrogen bonding preferences with nucleotides. For example, a protonated histidine may serve as a hydrogen bond donor for a hydrogen bond accepting thymine. However, when deprotonated at higher pH a deprotonated histidine may serve as a hydrogen bond acceptor for an adenine donor (Fig. 1F). Guanine and cytosine can be either a hydrogen bond donor or acceptor depending on the orientation of the nucleotide relative to the protein binding residue.

### SELEX-seq reveals pH-dependent binding preferences of FOXC2 *in vitro*

**Figure 2.**
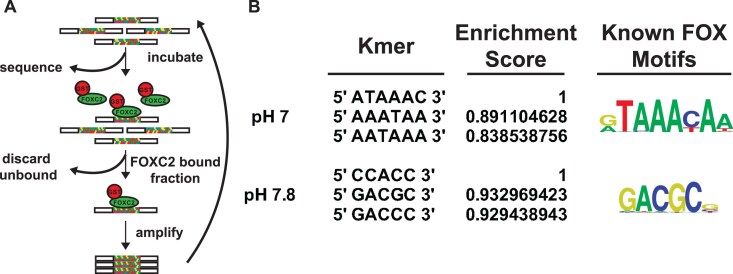
SELEX reveals pH-dependent differences in DNA-binding sequences for FOXC2. (**A**) Design of SELEX assay. (**B**) Most enriched sequences in SELEX-seq after four rounds of enrichment, each at pH 7.0 or 7.8 compared with known FOX consensus motifs from Nakagawa *et al.* [65].

To test our hypothesis, we used SELEX-seq as an unbiased approach to identify pH-dependent DNA binding motifs from a randomized library (Fig. 2A), using a purified recombinant FOXC2 DBD expressed as a GST fusion protein (Supplementary Fig. S2) at pH 7 and pH 7.8. We used FOXC2 because it is the only FOX family member with a crystal structure shown in complex with DNA that contains a single histidine (His122) in the DBD (Supplementary Fig. S1). Additionally, we limited our screen to pH 7 and 7.8 as representative pH values near the physiological limits of a cell [63, 64]. After four rounds of selection at pH 7, we find that the top three most enriched sequences are similar to the canonical Forkhead Primary (FkhP) motif ATAAACA (Fig. 2B). In contrast, at pH 7.8 the most enriched motif CCACC (SELEX1) is a prospective novel FOX consensus motif while the second most enriched sequence is the known FOX alternative consensus motif FHL [65] GACGC and is different than top hits at pH 7 (Fig. 2B). Further, neither SELEX1 nor FHL are detectably enriched at pH 7 while FkhP is minimally enriched at pH 7.8 (Supplementary File S1). These data suggest there are pH-dependent preferences in known FOX DNA-binding motifs for FOXC2.

### FOX family transcription factors have higher affinity for an FkhP motif at lower pH

**Figure 3.**
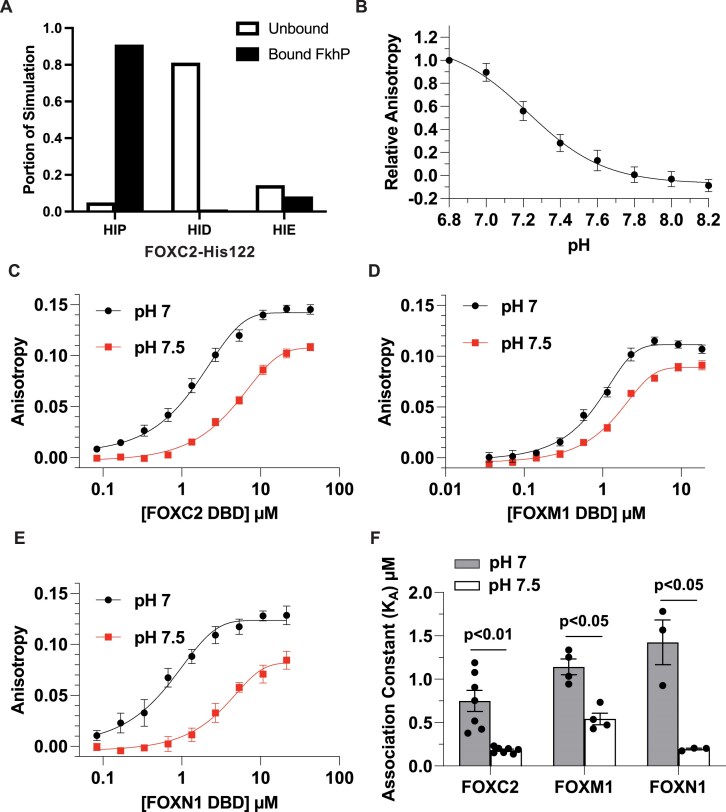
Binding of FOX family proteins to an FkhP sequence is pH-dependent with higher affinity at lower pH. (**A**) CpHMD simulations with FOXC2-His122 double protonated (HIP), protonated at delta nitrogen (HID), or protonated at epsilon nitrogen (HIE). Distribution of protonation states in simulations of unbound FOXC2 is in white bars and bound to an FkhP in black bars for each protonation state. (**B**) pH titration of recombinant FOXC2 binding to an FkhP sequence (*n* = 4). (**C**–**E**) Binding curves at pH 7.0 and 7.5 for FOXC2 (*n* = 7) (**C**), FOXM1 (*n* = 4) (**D**), and FOXN1 (*n* = 3) (**E**). (**F**) Association constants for FOXC2, FOXM1, and FOXN1 binding to an FkhP sequence at pH 7.0 and 7.5 calculated from binding curves. Data are means ± s.e.m. of at least three separate measurements with two–three independent recombinant protein preparations. Statistical analysis by Student’s paired *t*-test.

We tested the predicted protonation state of FOXC2-His122 when bound to the SELEX identified FkhP sequence *in silico* using CpHMD. Our data suggest that when FOXC2 is bound to the FkhP sequence, His122 is predicted to be doubly protonated in 91% of the simulation. Single protonated populations at the delta or epsilon nitrogen for FOXC2 in complex with DNA are observed only in 1% and 8% of the simulation, respectively. In contrast, when FOXC2 is in the unbound state His122 is predicted to be preferentially singularly protonated at the delta or epsilon nitrogen, 81% and 14% of the simulation respectively, while only doubly protonated in 5% (Fig. 3A). In addition to CpHMD at fixed pH, we calculated the p*K*a of titratable residues in the FOXC2 DNA-bound complex (PDB: 6AKO) and in an unbound FOXC2 model generated from the same structure using DelPhipKa web server [49, 50]. This approach approximates conformational changes associated with changes in protonation state through a Gaussian-based dielectric smooth function and allows to estimate the p*K*a of titratable residues across a range of pH values. We obtain consistent results that show a stabilization of protonated His122 when bound in complex with DNA (Supplementary Fig. S3A) and a downshifted predicted p*K*a for unbound FOXC2-His122 (Supplementary Fig. S3B). Together, these data suggest that FOXC2-His122 is a putative pH-sensing residue predicted to bind the canonical FkhP sequence when protonated at pH 7 in agreement with findings from SELEX-seq.

We next asked whether binding of FOX family proteins to the canonical FkhP sequence has higher affinity at lower pH *in vitro*, as predicted by both SELEX and CpHMD simulations. We first tested this prediction for FOXC2 by using fluorescence anisotropy with GST-FOXC2-DBD and a 5′ 6-FAM-labeled FkhP sequence. A pH titration of FOXC2 at sub-saturation protein concentration of 2.3 μM reveals that the overall affinity for the FkhP sequence decreases linearly within the cellular pH range between pH 7.0 to 7.8 with no observable change in binding above pH 7.8 at this protein concentration (Fig. 3B). We next determined the association constant (*K*_A_) for FOXC2 to the FkhP sequence at pH 7 and 7.5 and find that the binding affinity of FOXC2 for the FkhP sequence is significantly greater at pH 7.0 (*K*_A_: 0.75 ± 0.12 μM) compared with 7.5 (0.18 ± 0.02 μM) (Fig. 3C and F). We also asked whether other FOX family members have higher affinity binding to the FkhP sequence at lower pH. Using GST fusions of DBD sequences (Supplementary Fig. S2B and C), we find higher affinity binding for FOXM1 at pH 7.0 (*K*_A_: 1.1 ± 0.11 μM) compared with pH 7.5 (0.54 ± 0.08 μM) (Fig. 3D and F) and for FOXN1 at pH 7 (*K*_A_: 1.4 ± 0.31 μM) compared with pH 7.5 (0.19 ± 0.01 μM) (Fig. 3E and F). These findings indicate that binding of three FOX family members, FOXC2, FOXM1, and FOXN1, to the FkhP sequence is pH-dependent, with higher affinity binding at pH 7.0 compared with pH 7.5.

### His122 of FOXC2 is necessary for pH-dependent binding to the FkhP sequence

**Figure 4.**
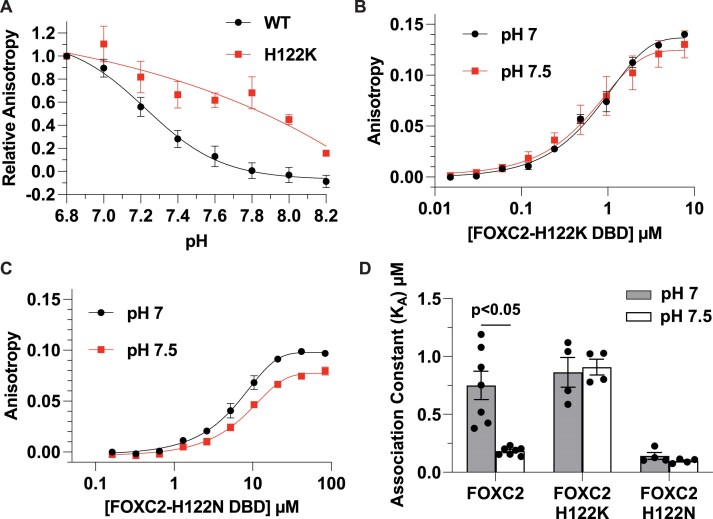
pH-dependent binding of FOXC2 to an FkhP sequence is dependent on His122. (**A**) pH titration of FOXC2-WT and -H122K (*n* = 3) for binding an FkhP sequence. (**B** and **C**) Binding curves of predicted pH-independent mutants FOXC2-H122K (*n* = 4) (B) and -H122N (*n* = 4) (C). (**D**) Association constants for FOXC2-WT, -H122K and -H122N calculated from binding curves. Data are means ± s.e.m. of at least three separate measurements with two–three independent protein preparations and statistical analysis by Tukey–Kramer HSD test.

To determine the significance of the conserved histidine for pH-regulated binding to the FkhP sequence we focused on FOXC2-His122 because it is the only histidine in the FOXC2 DBD. We used site-directed mutagenesis, first testing a His122Lys substitution with the prediction that lysine with a p*K*a of > 10 in solution would be constitutively charged within the cellular pH range and a hydrogen bond donor analogous to a protonated histidine. We find that FOXC2-H122K has strong relative binding affinity for the FkhP sequence between pH 6.8–8 (Fig. 4A). Further, when we performed a FOXC2-H122K protein titration,we find that the affinity at pH 7.0 (*K*_A_: 0.86 ± 0.15 μM) is similar to FOXC2-WT at pH 7.0 but pH-independent with no significant difference at pH 7.5 (*K*_A_: 0.91 ± 0.08 μM) (Fig. 4B and D). We also tested a His122Asn substitution, with the prediction that an asparagine would mimic a deprotonated histidine and have lower affinity and pH-independent binding to the FkhP sequence. Of significance, a naturally occurring FOXC2-H122N mutation is reported in lung cancers [66]. Our data confirm that the binding affinity of a FOXC2-H122N DBD is lower compared with WT at pH 7.0 and similar to WT at pH 7.5 but also pH-independent with no difference in affinity at pH 7.0 (K_A_: 0.14 ± 0.03 μM) compared with pH 7.5 (K_A_: 0.10 ± 0.01 μM) (Fig. 4C and D).

We also tested the importance of the conserved His287 in FOXM1 for pH-dependent DNA binding by using a His287Lys substitution. The affinities for FOXM1-H287K at pH 7 (*K*_A_: 4.1 ± 0.81 μM) and pH 7.5 (*K*_A_: 1.8 ± 0.27 μM) are both greater compared with FOXM1-WT at these pH values; however, binding is still pH-dependent (Supplementary Fig. S4A and B). To determine why FOXM1-His287 is the not the sole determinant for pH-dependent binding, we used CpHMD simulations to sample five histidine residues in the FOXM1-DBD. Our results predict that the protonation state of His269 (blue), His275 (cyan), and His311 (green) does not affect DNA binding. In contrast, both His287 (orange) and the proximal His292 (yellow) are predicted to prefer the DNA bound state when protonated and unbound state when neutral (Supplementary Fig. S3). Together, these data indicate that His122 is necessary for pH-dependent binding of FOXC2, but in FOX family proteins with more than one histidine in the DBD, the conserved histidine may contribute to but not exclusively confer pH-dependent DNA binding.

### FOXC2 has pHi-dependent activity in cells with greater activity for an FkhP reporter at lower pHi

**Figure 5.**
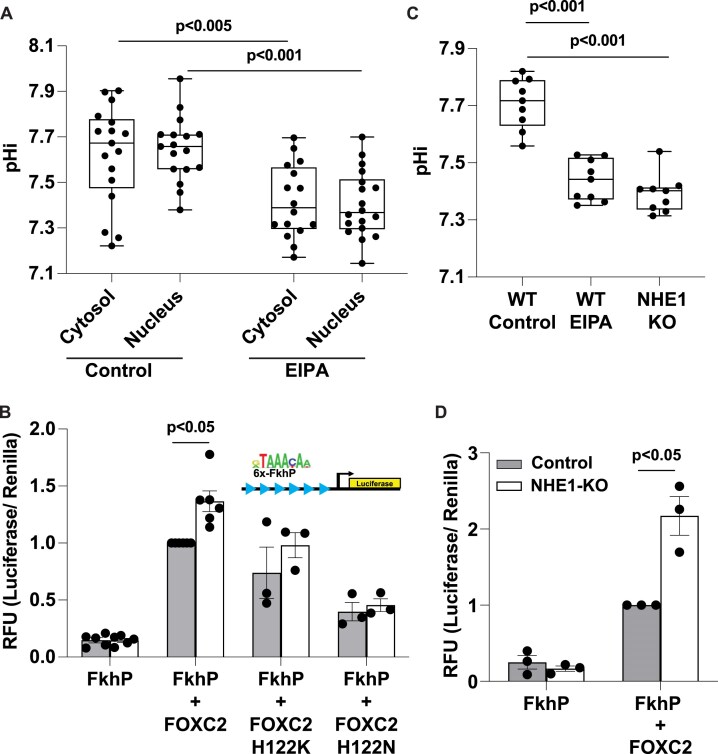
FOXC2 has pH-dependent transcriptional activity in cells determined by His122. (**A**) The cytosolic and nuclear pH of MDA-MB-436 cells in the absence (Control) and presence of EIPA (10 mM, 24 h). Box plots show median, first and third quartile, with whiskers extending to observations within 1.5 times the interquartile range, and individual data points representing an individual cell and including data from three separate cell preparations. Statistical analysis by Tukey–Kramer HSD test. (**B**) Activity of FOXC2-WT (*n* = 6), -H122K (*n* = 3), and -H122N (n = 3) determined using a 6X-FkhP luciferase reporter (insert) MDA-MB-436 cells in the absence (Control; pHi ∼7.7) and presence of EIPA (pHi ∼7.4) cells. Data are means ± s.e.m of three to four separate cell preparations with statistical analysis by Tukey–Kramer HSD test. (**C**) pHi of MDA-MB-436 cell populations, including WT in the absence (Control) and presence of EIPA (10 mM, 24 h) and CRISPR–Cas9-generated deletion of NHE1 (NHE1-KO). Box plots and statistical analysis as described in (A) from three separate cell preparations. Each data point represents the pHi value per well as a population of cells. (**D**) Activity of wild-type FOXC2 determined using a 6X-FkhP luciferase reporter in MDA-MB-436 cells, including WT in the controls and NHE1-silenced cells (*n* = 3). Data are means ± s.e.m of three separate cell preparations with statistical analysis by Student’s paired *t*-test.

We next asked whether pHi regulates FOXC2 activity for an FkhP sequence in cells by using a luciferase assay with MDA-MB-436 clonal human breast cancer cells. Imaging MDA-MB-436 cells loaded with the pH-sensitive dye SNARF, we find a relatively high pHi of 7.68 ± 0.21, which is common in cancer cells [13, 14] that is decreased to pHi 7.42 ± 0.04 in the presence of EIPA (10 μM, 24 h), a selective pharmacological inhibitor of the plasma membrane H + extruder NHE1 (Fig. 5A and Supplementary Fig. S4A). Imaging also revealed that pHi and nuclear pH are similar in control cells and cells treated with EIPA (Fig. 5A). In MDA-MB-436 cells transfected with a 6x-FkhP repeat luciferase reporter containing a minimal promoter (minP) (Fig. 5B, insert) in the absence of heterologous expressed FOXC2, there is minimal basal reporter activity with no significant difference between controls and cells treated with EIPA (Fig. 5B). In cells transfected with FOXC2-WT, however, there is a significantly greater relative reporter signal in the presence compared with the absence (control) of EIPA (Fig. 5B). We also tested activity of mutant FOXC2-H122K and FOXC2-H122N in MDA-MB-436 cells with the 6x-FkhP repeat luciferase reporter. We find that both mutant proteins have measured activity that is pH-independent (Fig. 5B), consistent with their pH-independent binding affinities for the FkhP sequence determined *in vitro* by fluorescence anisotropy (Fig. 4B–D). Although we find pH-independent luciferase activity in cells expressing FOXC2-H122K and FOXC2-H122N, the overall activity of FOXC2-H122K was lower than WT and lower than expected based on the observed high affinity *in vitro*. This difference may be due to a lower relative abundance of heterologous expressed FOXC2-H122K compared with WT (Supplementary Fig. S5C) or changes to tertiary structure of the full-length mutant protein compared with the shorter DBD used for *in vitro* binding measurements.

To lower pHi, we added EIPA 8 h after transfecting with FOXC2 and the luciferase reporter. As an alternative approach to eliminate a time delay, we generated MDA-MB-436 cells with CRISPR–Cas9 NHE1-KO. Using cell populations loaded with the pH-sensitive dye BCECF, we see a lower pHi of 7.39 ± 0.03 with NHE1-KO compared with a pHi of 7.71 ± 0.03 in control parental cells (Fig. 5C). Additionally, we confirmed loss of NHE1 activity in WT MDA-MB-436 cells treated with EIPA and in NHE1-KO cells by showing no pHi recovery in a nominally HCO_3_-free HEPES buffer from an NH_4_Cl-induced acid load compared with pHi recovery of WT cells (Supplementary Fig. S4B), which is an index of NHE1-dependent H + extrusion. With NHE1-KO, like with EIPA-treated WT cells, co-expression of the reporter and FOXC2 results in a >2-fold increase in activity compared with activity in parental controls (Fig. 5D) and in the absence of FOXC2 the reporter signal is minimal and pH-independent (Fig. 5D). Further, we confirmed these results are not due to differential FOXC2 expression in WT compared with NHE1-KO cells (Supplementary Fig. S5D). Together, these data using two different approaches to lower pHi, pharmacological and genetic, show that FOXC2 activity for an FkhP reporter in cells is regulated by pHi, with greater activity at pHi 7.4 compared with 7.7 likely mediated by His122.

### Fluorescence dual reporter reveals pH-dependent preferences for FOXN1 for FkhP and FHL motifs

Our SELEX-seq data (Fig. 2) suggests at higher pH FOXC2 preferentially binds to a previously reported FOX transcription factor binding motif, FHL [43, 65]. To confirm these findings we tested binding of the DBD of FOXC2, FOXM1, and FOXN1 to a 5′ 6-FAM labeled FHL sequence. However, we did not observe measurable binding at pH 7, 7.5, or 7.8. We also did not observe measurable binding when we lengthened the FHL sequence to a 2× repeat (cGACGCGACGC) to match the sequence length (11 bp) of the FkhP oligo nor a (30 bp) FHL sequence or when we reduced buffer osmolarity to 50 mM as previously reported for FOXN1-FHL anisotropy assays [67].

**Figure 6.**
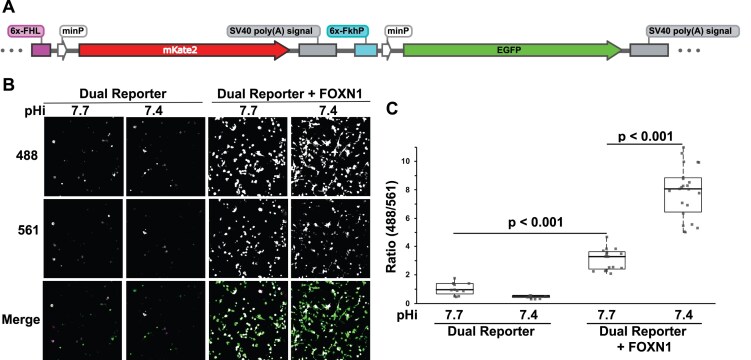
Fluorescent dual reporter shows pH-dependent differences in FOXN1 activity for FkhP and FHL sequences. (**A**) Design of dual reporter with a 6x FHL driving expression of mKate and a 6x FkhP driving expression of eGFP. (**B**) Images at Ex488, Ex561 and merged of MDA-MB-436 cells expressing the dual reporter in the absence and presence of heterologous expressed wild-type full-length FOXN1 at pHi 7.7 (WT cells) and pHi 7.4 (NHE1-KO cells). (**C**) Quantified fluorescent ratios (Ex488/Ex561) as per images shown in (**B**) for the dual reporter in the absence and presence of heterologous expressed wild-type full-length FOXN1 at the indicated pHi values. Box plots show median, first and third quartile, with whiskers extending to observations within 1.5 times the interquartile range, and individual data points representing values from a single image with at least five images analysed in three separate cell preparations. Statistical analysis by Tukey–Kramer HSD test.

We, therefore, tested pHi-dependent preference for binding the FHL motif in cells. We generated a novel fluorescent dual reporter in which a 6x FHL repeat drives expression of mKate (561) and a 6x FkhP repeat drives expression of enhanced green fluorescent protein (eGFP) (488) (Fig. 6A). We find minimal basal reporter activity when we transfect MDA-MB-436 cells with only the dual reporter and the fluorescence ratio intensity at 488/561 is not different at pHi 7.4 and 7.7 (Fig. 6B and C). However, when we co-transfect the reporter with full-length FOXN1 we find there are significant pHi-related differences in the 488/561 ratio, with a greater ratio at pHi 7.4 compared with a lower ratio at 7.7, suggesting preferential binding and activity of FOXN1 to FkhP at low pHi and FHL at higher pHi. Further, we confirmed the higher 488/561 ratio at pHi 7.4 compared with 7.7 is due to both a higher mean fluorescence for eGFP and a concomitant lower mean fluorescence for mKate (Supplementary Fig. S6A and B). These findings are consistent with a report that FOXN1 binds FHL with higher affinity than FkhP at pH 7.5 [67] and demonstrate that in cells, FOXN1 has greater binding and activity at an FkhP promoter at lower pHi but preference for an FHL promoter at higher pHi. For unclear reasons, however, with heterologous expression of full-length FOXC2-WT we do not observe changes in reporter fluorescence intensities at 488 or 561 nm compared with basal intensities, despite our findings with FOXC2 using a luciferase reporter with a 6x-FkhP repeat (Fig. 5B).

### ChIP-seq reveals pH-regulated differences in DNA binding motif preferences

**Figure 7.**
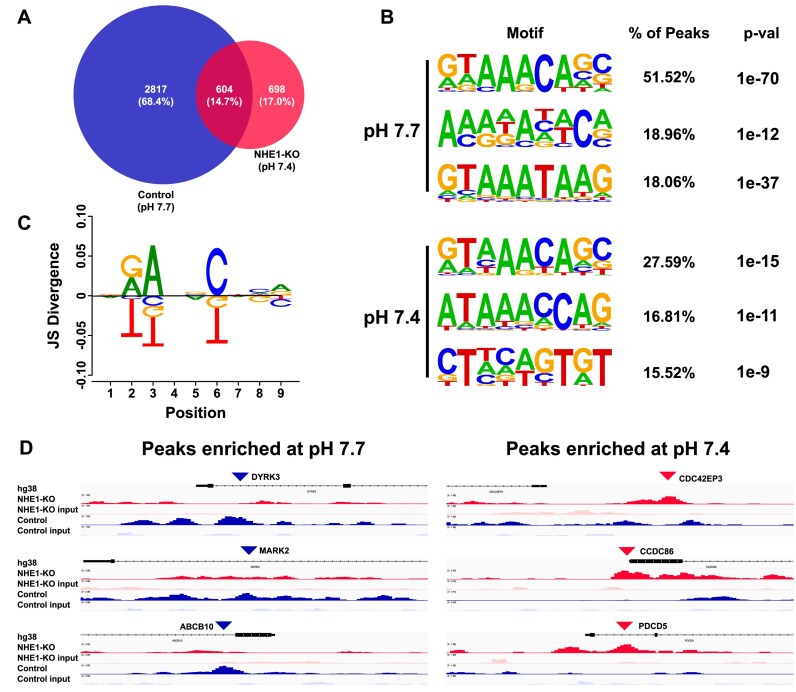
ChIP-seq reveals pH-regulated differences in binding motif preferences and gene enrichment. (**A**) Peak count of ChIP peaks in control (pH 7.7) (blue) or NHE1-KO cells (pH 7.4) (red). Overlap shows number of shared peaks between both control and NHE1-KO. Peaks are from three separate cell preparations. (**B**) Top three HOMER identified motifs uniquely enriched in either control peaks (2817) or NHE1-KO peaks (698) ranked by the percentage of peaks the motif is observed. (**C**) Positional nucleotide comparison of top enriched motif at pH 7.7 (top) compared to pH 7.4 (bottom). (**D**) Select peaks enriched at pH 7.7 (blue) and 7.4 (red). Each track is set to 5 kb and are representative replicates for ChIP (dark) and matched input (light).

We next asked whether FOXC2 genome-wide binding is pHi-dependent with differences in both DNA-motif preferences and gene enrichment. Using MDA-MB-436 control (pHi 7.7) or NHE1-KO (pHi 7.4) cells transiently transfected with WT Flag-FOXC2, we performed ChIP-seq and determined peaks enriched in each condition. While we identified 604 shared peaks between conditions, we also find there are 2817 and 698 peaks unique to control (blue) or NHE1-KO (red) cells, respectively (Fig. 7A and Supplementary File S2). We used HOMER findMotifsGenome.pI [54] to identify *de novo* enriched DNA motifs within 200 bp of peak coordinates unique to control or NHE1-KO cells. Results were ranked by percentage of peaks with distinct motifs. Although we expected enrichment of FHL-like motifs for control cells (pHi 7.7) similar to SELEX-seq, we find the first (51.52%) and third (18.06%) most enriched motifs are highly similar to FkhP. However, for these motifs we find thymine is notably depleted in the second nucleotide position where FOXC2-His122 is predicted to make a hydrogen bond as our model predicts for a deprotonated histidine. In contrast, for NHE1-KO cells (pHi 7.4), we find the first (27.59%) and second (16.81%) most enriched motifs are indeed FkhP with high conservation of a thymine in the second position as expected for binding a protonated histidine. While the second and third motifs identified at pHi 7.7 (18.96%) and 7.4 (15.52%), respectively, do not resemble known FOX consensus sites, consistent with our other identified motifs we find the second position is either thymine depleted or enriched as expected (Fig. 7B). To visually compare the most enriched motif at pHi 7.7 and 7.4 we used DiffLogo [55], which plots motifs as positional weighted nucleotide probabilities with larger nucleotide logos representing greater differences. While most positions of each motif are highly similar, we find in the second position that guanine and adenine are selectively enriched at pHi 7.7 and thymine is enriched at pHi 7.4 (Fig. 7C). Next, we visually examined representative select peaks uniquely enriched in gene promoter regions of control (blue tracks) or NHE1-KO (red tracks) at a 5-kb scale using the Integrative Genome Viewer [56]. For control cells we find peaks in promoter regions for genes DYRK3, MARK2, and ABCB10, which are known to promote cancer behaviours including migration, proliferation, inhibition of apoptosis, and metabolic reprogramming [68–71], consistent with a higher pHi enabling cell migration [4–9] and proliferation [1–3]. In contrast, for NHE1-KO cells we find peaks in promoters for representative genes CDC42EP3 and CCD86, which are known FOX target genes [72] and the pro-apoptotic gene PDCD5 [73–75]. Taken together, these results suggest pHi dependent differences in FOXC2 binding to distinct DNA motifs and gene promoters as well as preferential binding to thymine at pHi 7.4 compared with pHi 7.7.

### RNA-seq identifies pH-dependent differences in gene expression and suggested binding motifs

**Figure 8.**
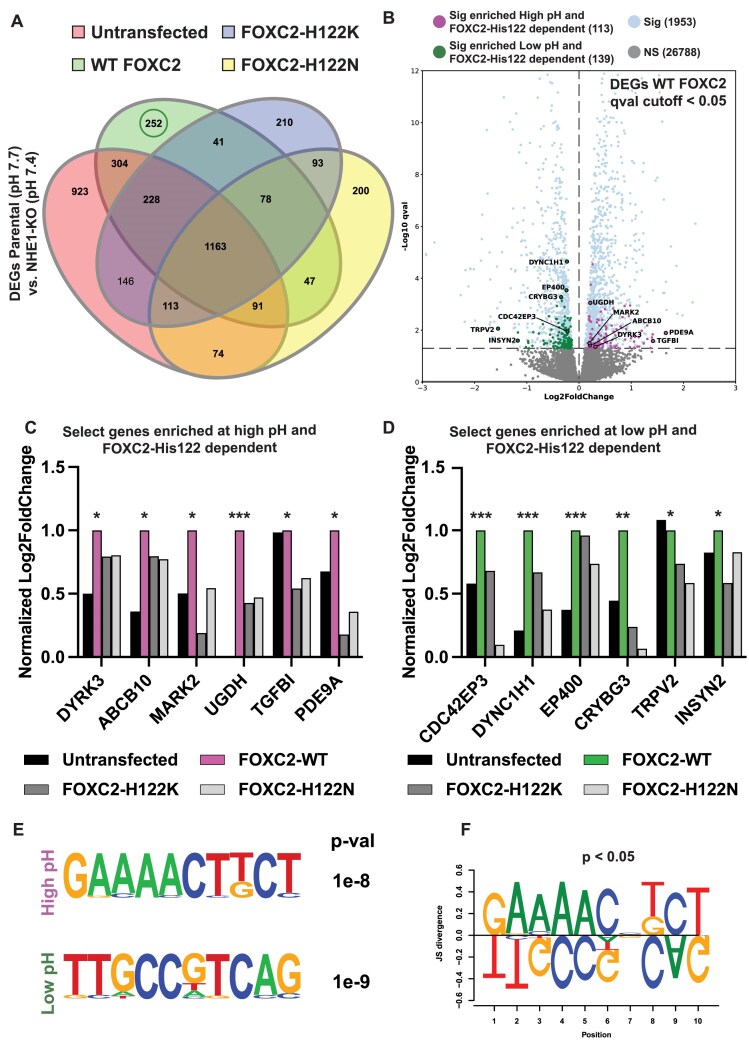
RNA-seq reveals pH-regulated and FOXC2-His122-dependent differences in gene expression and enriched binding motifs. (**A**) DEG sets between control (pH 7.7) and NHE1-KO (pH 7.4) MDA-MB-436 cells in untransfected (red), overexpressing FOXC2-WT (green), FOXC2-H122K (blue), or FOXC2-H122N (yellow). (**B**) Volcano plots of DEGs (qval < 0.05) in control compared with NHE1-KO MDA-MB-436 cells overexpressing FOXC2-WT. Each dot represents a single gene with pH- and FOXC2-His122 dependent genes enriched at high (magenta) versus low (green) pH and all other DEGs (blue). (**C** and **D**) Log_2_ fold change of enriched genes at pH 7.7 (C) or pH 7.4 (D) normalized to WT-FOXC2 for each gene with asterisks indicating qval where ***, **, and * are *q* < 0.001, 0.005, and 0.05, respectively. (**E**) Common promoter elements in genes enriched at high and low pH identified by HOMER. (**F**) Position weight matrix comparison of promoter elements.

As an additional unbiased approach to test pHi regulated differences in FOXC2 activity, we used RNA-seq to determine DEGs and enriched promoter elements dependent on both pHi and FOXC2-His122. To establish genes that are both pHi and FOXC2-His122 dependent we performed bulk RNA-seq in control (pHi 7.7) and NHE1-KO (pHi 7.4) MDA-MB-436 cells that were either untransfected, transiently expressing recombinant FOXC2-WT, or transiently expressing pH-independent mutants FOXC2-H122K or FOXC2-H122N (Supplementary Files S3–6). We find that in cells transfected with FOXC2-WT there are 2204 DEGs between control and NHE1-KO cells. However, we find that only 252 of these DEGs (green circle) are both pHi and FOXC2-His122 dependent due to lack of differential expression of these genes in untransfected or with pH-independent mutants (Fig. 8A). Further, we determined which of the pHi- and FOXC2-His122 dependent DEGs were enriched at a higher pHi of 7.7 (controls) compared with lower pHi of 7.4 (NHE1-KO). Of the 252 DEGs, we find 113 are significantly enriched in controls at pHi 7.7 while 139 are enriched in NHE1-KO cells at pHi 7.4 (Fig. 8B). Selected genes enriched at higher pHi were based on genes also identified with ChIP-seq peaks in control (DYRK3, ABCB10, and MARK2) (Fig. 7D), high *q*-value and the known FOXC2 target (UGDH) [76], or high fold-change (PDE9A and TGFBI), which promote epithelial-to-mesenchymal transition (EMT), cell migration, metastasis, proliferation, and angiogenesis (Fig. 8C) [68–71, 76–88], consistent with a higher pHi enabling EMT [64, 89], cell migration [4–9], and proliferation [1–3]. In contrast, genes enriched at pHi 7.4 include CDC42EP3, which we also find to be selectively enriched in NHE1-KO cells with ChIP-seq (Fig. 7D), DYNC1H1, and INSYN2, which are not known tumor promoting genes, as well as EP400, CRYBG3, and TRPV2, which promote tumor suppression, cell-cycle arrest, senescence, DNA-damage response, and apoptosis (Fig. 8D) [90–97]. Together these results highlight there are pHi- and FOXC2-His122-dependent differences in gene expression.

To determine whether promoter elements of the DEGs enriched at pHi 7.7 compared with 7.4 are different, we used HOMER findMotif.pl [54], which does reveal different motifs at higher compared with lower pHi (Fig. 8E and F). Similar to findings from ChIP-seq, at the second nucleotide, motifs enriched at pHi 7.7 contain either a cytosine or adenine while motifs enriched at pHi 7.4 contain an invariant thymine as predicted (Fig. 8F). However, using fluorescence anisotropy measurements we again do not observe significant binding of FOXC2 to these HOMER identified motifs at either pH 7 or 7.5, suggesting like in SELEX-seq identified motifs, co-factors may be necessary for high affinity binding, which requires further study.

## Discussion

We report an underappreciated role of pH dynamics in regulating transcription factor-DNA binding selectivity. The concept is relevant to transcription factors with a histidine in the DBD that forms hydrogen bonds directly with DNA nucleotides and could apply to at least 85 transcription factors across multiple families, as shown in Fig. 1A and Supplementary Fig. S1. Our multidisciplinary approaches collectively identify distinct DNA-binding motif preferences for selective FOX family transcription factors, with a preference for binding thymine at lower pH and for binding AGC rich sequences at higher pH. We do not predict that pHi dynamics functions as a binary switch for DNA binding preference but rather acts as a coincidence regulator with other established mechanisms such as co-factor association, post-translational modifications like phosphorylation, and DNA accessibility through epigenetic modifications [98]. For the latter, select pH-sensing epigenetic readers and writers were recently shown to regulate chromatin accessibility [99, 100].

We confirm that three FOX family transcription factors, FOXC2, FOXM1, and FOXN1, bind the canonical FkhP DNA sequence (ATAAACA) with higher affinity at lower pH *in vitro* and for FOXC2 and FOXN1 also in cells. Additionally, for FOXC2, pH-dependent DNA binding and activity are conferred by a conserved His122. These data are consistent with our prediction that a protonated histidine at lower pH acts as a hydrogen bond donor for thymine, which is generally a hydrogen bond acceptor. Two previous reports with other FOX family members also indicate pH-regulated functions. Using FOXP2, Blane and Fanucchi [101] show that the protonation state of the conserved histidine in the DBD regulates protein tertiary shape and DNA-binding affinity for the FkhP motif, although their study included nonphysiological pH values as low as pH 5 and as high as pH 9. Using FOXP1, Medina *et al.* [102] show that the protonation state of a less conserved histidine exclusive to the FOXM/O/P subfamilies modulates domain swapping stability to regulate DNA-binding affinity. Collectively, these findings and our current study strongly support a pH-regulated binding of FOX family transcription factors to an FkhP motif with higher affinity binding at lower pH.

Our corollary prediction, that a deprotonated histidine acts as a hydrogen bond acceptor at higher pH to favor binding to an adenine or reduce its preference for thymine is supported by our SELEX-seq, ChIP-seq, and RNA-seq data. These three unbiased approaches reveal pH-dependent binding preferences with lower pH favoring binding to thymine and higher pH favoring binding to AGC rich sequences, or in the case of ChIP-seq, depleting the overwhelming preference for thymine. Although reduced, thymine may persist in motifs identified by ChIP-seq at higher pHi due to a fraction of FOXC2-His122 remaining protonated. This could be explained by the expected heterogeneity of protonation state at a pHi near the p*K*a of a residue or the potential that the p*K*a can be dramatically upshifted when FOXC2 is bound to cofactors in cells. Of note, while we consistently identify preference for FkhP with each unbiased approach at lower pH, the top identified motifs at higher pH are different as determined by SELEX-seq, ChIP-seq, and RNA-seq. Of note, however, both ChIP-seq and RNA-seq confirm that in cells, FOXC2 preferentially binds distinct gene promoters and regulates the expression of different genes at pHi 7.7 compared with pHi 7.4. Additionally, several enriched genes at higher pHi (DYRK3, ABCB10, and MARK2) and lower pHi CDC42EP3 were commonly identified by ChIP-seq and RNA-seq. At pHi 7.7, several of the unique enriched peaks and genes with higher expression dependent on pH-sensing by FOXC2-His122 are known tumor promoting genes [68–71, 76, 86, 87]. Further, peak enrichment and expression of several tumor suppressor genes enriched at pHi 7.4 are attenuated at pHi 7.7 [70, 74, 75, 90–97]. These results are consistent with previous findings that increased pHi promotes cancer cell behaviours including cell cycle progression, EMT, migration, and invasion [3, 13–15, 58, 64, 89, 103].

Although we could not confirm *in vitro* higher affinity binding at higher pH to GACGC identified by SELEX-seq that resembles a reported FOX FHL consensus motif [65], we show in cells using a fluorescent dual reporter we developed, a preference of FOXN1 for binding a FkhP motif at lower pHi and for an FHL motif at higher pHi. Two possibilities could account for our inability to confirm *in vitro* binding of FOXC2 or FOXN1 to the motifs identified by SELEX-seq at higher pH. First, PCR amplification steps in SELEX-seq may overrepresent overall binding affinity of FOXC2. Second, the short 5 base pair sequences or 2x repeats might not been sufficient for FOXC2 binding without flanking nucleotides present in the SELEX library.

Our findings suggest a possible mechanism for how pHi directly regulates gene expression through pH-sensing of transcription factors and further highlights pHi as a signaling mechanism regulating protein electrostatics for changes in cell behaviours. Further, our RNA-seq results underscore how relatively small changes in pHi can lead to large scale changes in a gene expression as well as predicted DNA motif-binding preferences. These findings are consistent with our previous reports on pH sensing by endogenous proteins with small changes in the cellular pH range conferring cell behaviours, including stability of β-catenin by pH-regulated binding to the E3 ligase β-TrCP regulating epithelial cell integrity [30], activity of the focal adhesion kinase FAK through in-*cis* conformational changes conferring cell substrate adhesion dynamics [7], and the affinity of talin for binding F-actin in controlling cell migration [6].

The concept of histidine titration conferring nucleotide-binding selectivity can be applied to a broader scope. First, although our study focused exclusively on FOX family members, pH-dependent binding of transcription factors to DNA may be a relevant to transcription factors in other families such as KLF, SOX, and MITF/MYC/MAX that contain a conserved histidine in the DBD, which in available structures forms a hydrogen bond with nucleotides. For the SOX family, Sry and SOX30 are the only members that lack a nucleotide-binding histidine, and instead contain a cognate arginine and asparagine, respectively. Future studies could use DBD swapping with Sry and SOX30, which cannot be applied to FOX proteins that contain an invariant histidine. Second, the concept can be applied to cell behaviours regulated by pHi dynamics that include changes in gene expression, most notably stem cell differentiation and lineage specification [10–12]. Third, we propose that histidine titration conferring nucleotide-binding selectivity could in part determine how different co-expressed members of transcription factor families with highly conserved DNA-binding domains recognize distinct DNA sequences and are used reiteratively to regulate diverse target genes and disparate cell behaviours, which are unresolved questions for understanding developmental processes. Finally, beyond transcription factors, some RNA-binding proteins contain a critical nucleotide-binding histidine [104].

Based on our current findings and applications for a broader scope, we propose that pH dynamics is an understudied mechanism regulating nucleotide binding by proteins with functionally critical histidine residues. Moreover, we highlight how pHi regulated electrostatics can affect protein functions for diverse cell behaviours for a more complete understanding of both development and disease.

## Supplementary Material

gkaf474_Supplemental_Files

## Data Availability

The data underlying this article are available in Gene Expression Omnibus at (https://www.ncbi.nlm.nih.gov/geo/) and can be accessed with accession codes GSE270316 for SELEX-seq, GSE270232 for RNA-seq, and GSE293822 for ChIP-seq.

## References

[1] Putney LK, Barber DL Na-H exchange-dependent increase in intracellular pH times G2/M entry and transition. J Biol Chem. 2003; 278:44645–9. 10.1074/jbc.M308099200. 12947095

[2] Flinck M, Kramer SH, Schnipper J et al. The acid–base transport proteins NHE1 and NBCn1 regulate cell cycle progression in human breast cancer cells. Cell Cycle. 2018; 17:1056–67. 10.1080/15384101.2018.1464850. 29895196 PMC6110587

[3] Spear JS, White KA Single-cell intracellular pH dynamics regulate the cell cycle by timing the G1 exit and G2 transition. J Cell Sci. 2023; 136:jcs260458 10.1242/jcs.260458. 37133398 PMC10281514

[4] Denker SP, Barber DL Cell migration requires both ion translocation and cytoskeletal anchoring by the Na-H exchanger NHE1. J Cell Biol. 2002; 159:1087–96. 10.1083/jcb.200208050. 12486114 PMC2173980

[5] Frantz C, Barreiro G, Dominguez L et al. Cofilin is a pH sensor for actin free barbed end formation: role of phosphoinositide binding. J Cell Biol. 2008; 183:865–79. 10.1083/jcb.200804161. 19029335 PMC2592832

[6] Srivastava J, Barreiro G, Groscurth S et al. Structural model and functional significance of pH-dependent talin-actin binding for focal adhesion remodeling. Proc Natl Acad Sci USA. 2008; 105:14436–41. 10.1073/pnas.0805163105. 18780792 PMC2532973

[7] Choi CH, Webb BA, Chimenti MS et al. pH sensing by FAK-His58 regulates focal adhesion remodeling. J Cell Biol. 2013; 202:849–59. 10.1083/jcb.201302131. 24043700 PMC3776353

[8] Martin C, Pedersen SF, Schwab A et al. Intracellular pH gradients in migrating cells. Am J Physiol-Cell Physiol. 2011; 300:C490–5. 10.1152/ajpcell.00280.2010. 21148407

[9] Clement DL, Mally S, Stock C et al. PDGFRα signaling in the primary cilium regulates NHE1-dependent fibroblast migration via coordinated differential activity of MEK1/2-ERK1/2-p90RSK and AKT signaling pathways. J Cell Sci. 2013; 126:C490–5. 10.1242/jcs.116426 PMC4481637 23264740

[10] Benitez M, Tatapudy S, Liu Y et al. Drosophila anion exchanger 2 is required for proper ovary development and oogenesis. Dev Biol. 2019; 452:127–33. 10.1016/j.ydbio.2019.04.018. 31071312 PMC6592727

[11] Liu Y, Reyes E, Castillo-Azofeifa D et al. Intracellular pH dynamics regulates intestinal stem cell lineage specification. Nat Commun. 2023; 14:3745 10.1038/s41467-023-39312-9. 37353491 PMC10290085

[12] Ulmschneider B, Benitez M, Azimova DR et al. Increased intracellular pH is necessary for adult epithelial and embryonic stem cell differentiation. J Cell Biol. 2016; 215:345–55. 10.1083/jcb.201606042. 27821494 PMC5100294

[13] Webb BA, Chimenti M, Jacobson MP et al. Dysregulated pH: a perfect storm for cancer progression. Nat Rev Cancer. 2011; 11:671–7. 10.1038/nrc3110. 21833026

[14] White KA, Grillo-hill BK, Barber DL Cancer cell behaviors mediated by dysregulated pH dynamics at a glance. J Cell Sci. 2017; 130:663–9. 10.1242/jcs.195297. 28202602 PMC5339414

[15] Swietach P, Boedtkjer E, Pedersen SF How protons pave the way to aggressive cancers. Nat Rev Cancer. 2023; 23:825–41. 10.1038/s41568-023-00628-9. 37884609

[16] Gillies RJ, Pilot C, Marunaka Y et al. Targeting acidity in cancer and diabetes. Biochim Biophys Acta. 2019; 1871:273–80. 10.1016/j.bbcan.2019.01.003. PMC6525044 30708040

[17] Hayata H, Miyazaki H, Niisato N et al. Lowered extracellular pH is involved in the pathogenesis of skeletal muscle insulin resistance. Biochem Biophys Res Commun. 2014; 445:170–4. 10.1016/j.bbrc.2014.01.162. 24502946

[18] Majdi A, Mahmoudi J, Sadigh-Eteghad S et al. Permissive role of cytosolic pH acidification in neurodegeneration: a closer look at its causes and consequences. J Neurosci Res. 2016; 94:879–87. 10.1002/jnr.23757. 27282491

[19] Harguindey S, Stanciu D, Devesa J et al. Cellular acidification as a new approach to cancer treatment and to the understanding and therapeutics of neurodegenerative diseases. Semin Cancer Biol. 2017; 43:157–79. 10.1016/j.semcancer.2017.02.003. 28193528

[20] Schönichen A, Webb BA, Jacobson MP et al. Considering protonation as a posttranslational modification regulating protein structure and function. Annu Rev Biophys. 2013; 42:289–314. 10.1146/annurev-biophys-050511-102349. 23451893 PMC4041481

[21] Sun S, Poudel P, Alexov E et al. Electrostatics in computational biophysics and its implications for disease effects. Int J Mol Sci. 2022; 23:10347 10.3390/ijms231810347. 36142260 PMC9499338

[22] Isom DG, Castañeda CA, Cannon BR et al. Large shifts in pK a values of lysine residues buried inside a protein. Proc Natl Acad Sci USA. 2011; 108:5260–5. 10.1073/pnas.1010750108. 21389271 PMC3069169

[23] Karp DA, Stahley MR, García-Moreno EB Conformational consequences of ionization of lys, asp, and glu buried at position 66 in staphylococcal nuclease. Biochemistry. 2010; 49:4138–46. 10.1021/bi902114m. 20329780 PMC3373020

[24] Kazyken D, Lentz SI, Wadley M et al. Alkaline intracellular pH (pHi) increases PI3K activity to promote mTORC1 and mTORC2 signaling and function during growth factor limitation. J Biol Chem. 2023; 299:105097 10.1016/j.jbc.2023.105097. 37507012 PMC10477693

[25] Morales Rodríguez LM, Crilly SE, Rowe JB et al. Location-biased activation of the proton-sensor GPR65 is uncoupled from receptor trafficking. Proc Natl Acad Sci USA. 2023; 120:e2302823120 10.1073/pnas.2302823120. 37722051 PMC10523530

[26] Webb BA, White KA, Grillo-Hill BK et al. A histidine cluster in the cytoplasmic domain of the Na-H exchanger NHE1 confers pH-sensitive phospholipid binding and regulates transporter activity. J Biol Chem. 2016; 291:24096–104. 10.1074/jbc.M116.736215. 27650500 PMC5104935

[27] Johnston RJ, Su LJ, Pinckney J et al. VISTA is an acidic pH-selective ligand for PSGL-1. Nature. 2019; 574:565–70. 10.1038/s41586-019-1674-5. 31645726

[28] Lu Y, Zuo P, Chen H et al. Structural insights into the conformational changes of BTR1/SLC4A11 in complex with PIP2. Nat Commun. 2023; 14:6157 10.1038/s41467-023-41924-0. 37788993 PMC10547724

[29] Onufriev AV, Alexov E Protonation and pK changes in protein-ligand binding. Quart Rev Biophys. 2013; 46:181–209. 10.1017/S0033583513000024. PMC4437766 23889892

[30] White KA, Esquivel M, Barber DL et al. β-Catenin is a pH sensor with decreased stability at higher intracellular pH. J Cell Biol. 2018; 217:3965–76. 10.1083/jcb.201712041. 30315137 PMC6219716

[31] Malevanets A, Chong PA, Hansen DF et al. Interplay of buried histidine protonation and protein stability in prion misfolding. Sci Rep. 2017; 7:882 10.1038/s41598-017-00954-7. 28408762 PMC5429843

[32] Wang W, Xi L, Xiong X et al. Insight into the structural stability of wild-type and histidine mutants in Pin1 by experimental and computational methods. Sci Rep. 2019; 9:8413 10.1038/s41598-019-44926-5. 31182777 PMC6557836

[33] Westermark P, Andersson A, Westermark GT Islet amyloid polypeptide, islet amyloid, and diabetes mellitus. Physiol Rev. 2011; 91:795–826. 10.1152/physrev.00042.2009. 21742788

[34] Xiang W, Menges S, Schlachetzki JCM et al. Posttranslational modification and mutation of histidine 50 trigger alpha synuclein aggregation and toxicity. Mol Neurodegen. 2015; 10:8 10.1186/s13024-015-0004-0. PMC4365527 25886189

[35] Putney LK, Barber DL Expression profile of genes regulated by activity of the Na–H exchanger NHE1. BMC Genomics. 2004; 5:46 10.1186/1471-2164-5-46. 15257760 PMC499544

[36] Casey JR, Grinstein S, Orlowski J Sensors and regulators of intracellular pH. Nat Rev Mol Cell Biol. 2010; 11:50–61. 10.1038/nrm2820. 19997129

[37] Lam EWF, Brosens JJ, Gomes AR et al. Forkhead box proteins: tuning forks for transcriptional harmony. Nat Rev Cancer. 2013; 13:482–95. 10.1038/nrc3539. 23792361

[38] Lee BK, Bhinge AA, Battenhouse A et al. Cell-type specific and combinatorial usage of diverse transcription factors revealed by genome-wide binding studies in multiple human cells. Genome Res. 2012; 22:9–24. 10.1101/gr.127597.111. 22090374 PMC3246210

[39] Wang Z, Yu T, Huang P Post-translational modifications of FOXO family proteins (Review). Mol Med Rep. 2016; 14:4931–41. 10.3892/mmr.2016.5867. 27779663

[40] Dai S, Qu L, Li J et al. Toward a mechanistic understanding of DNA binding by forkhead transcription factors and its perturbation by pathogenic mutations. Nucleic Acids Res. 2021; 49:10235–49. 10.1093/nar/gkab807. 34551426 PMC8501956

[41] Rohs R, Jin X, West SM et al. Origins of specificity in protein–DNA recognition. Annu Rev Biochem. 2010; 79:233–69. 10.1146/annurev-biochem-060408-091030. 20334529 PMC3285485

[42] Ibarra IL, Hollmann NM, Klaus B et al. Mechanistic insights into transcription factor cooperativity and its impact on protein–phenotype interactions. Nat Commun. 2020; 11:124 10.1038/s41467-019-13888-7. 31913281 PMC6949242

[43] Rogers JM, Waters CT, Seegar TCM et al. Bispecific forkhead transcription factor FoxN3 recognizes two distinct motifs with different DNA shapes. Mol Cell. 2019; 74:245–53. 10.1016/j.molcel.2019.01.019. 30826165 PMC6474805

[44] Oginuma M, Harima Y, Tarazona OA et al. Intracellular pH controls WNT downstream of glycolysis in amniote embryos. Nature. 2020; 584:98–101. 10.1038/s41586-020-2428-0. 32581357 PMC8278564

[45] Moparthi L, Koch S A uniform expression library for the exploration of FOX transcription factor biology. Differentiation. 2020; 115:30–6. 10.1016/j.diff.2020.08.002. 32858261

[46] Riley TR, Slattery M, Abe N et al. SELEX-seq: a method for characterizing the complete repertoire of binding site preferences for transcription factor complexes. Methods Mol Biol. 2014; 1196:255–78. 25151169 10.1007/978-1-4939-1242-1_16 PMC4265583

[47] Swails JM, York DM, Roitberg AE Constant pH replica exchange molecular dynamics in explicit solvent using discrete protonation states: implementation, testing, and validation. J Chem Theory Comput. 2014; 10:1341–52. 10.1021/ct401042b. 24803862 PMC3985686

[48] Peng Y, Alexov E Computational investigation of proton transfer, p*K*a shifts and pH-optimum of protein–DNA and protein–RNA complexes. Proteins. 2017; 85:282–95. 10.1002/prot.25221. 27936518 PMC9843452

[49] Wang L, Li L, Alexov E pKa predictions for proteins, RNAs, and DNAs with the gaussian dielectric function using DelPhi pKa. Proteins. 2015; 83:2186–97. 10.1002/prot.24935. 26408449 PMC4715546

[50] Wang L, Zhang M, Alexov E DelPhiPKa web server: predicting pK a of proteins, RNAs and DNAs. Bioinformatics. 2016; 32:614–5. 10.1093/bioinformatics/btv607. 26515825 PMC5963359

[51] Grillo-Hill BK, Webb BA, Barber DL Ratiometric imaging of pH probes. Methods Cell Biol. 2014; 123:429–48. 24974041 10.1016/B978-0-12-420138-5.00023-9

[52] Meima ME, Webb BA, Witkowska HE et al. The sodium–hydrogen exchanger NHE1 is an akt substrate necessary for actin filament reorganization by growth factors. J Biol Chem. 2009; 284:26666–75. 10.1074/jbc.M109.019448. 19622752 PMC2785354

[53] Hulsen T, de Vlieg J, Alkema W BioVenn—a web application for the comparison and visualization of biological lists using area-proportional Venn diagrams. BMC Genomics. 2008; 9:488 10.1186/1471-2164-9-488. 18925949 PMC2584113

[54] Heinz S, Benner C, Spann N et al. Simple combinations of lineage-determining transcription factors prime cis-regulatory elements required for macrophage and B cell identities. Mol Cell. 2010; 38:576–89. 10.1016/j.molcel.2010.05.004. 20513432 PMC2898526

[55] Nettling M, Treutler H, Grau J et al. DiffLogo: a comparative visualization of sequence motifs. BMC Bioinf. 2015; 16:387 10.1186/s12859-015-0767-x. PMC4650857 26577052

[56] Robinson JT, Thorvaldsdóttir H, Winckler W et al. Integrative genomics viewer. Nat Biotechnol. 2011; 29:24–26. 10.1038/nbt.1754. 21221095 PMC3346182

[57] Heberle H, Meirelles VG, da Silva FR et al. InteractiVenn: a web-based tool for the analysis of sets through Venn diagrams. BMC Bioinf. 2015; 16:169 10.1186/s12859-015-0611-3. PMC4455604 25994840

[58] Frantz C, Karydis A, Nalbant P et al. Positive feedback between Cdc42 activity and H+ efflux by the Na-H exchanger NHE1 for polarity of migrating cells. J Cell Biol. 2007; 179:403–10. 10.1083/jcb.200704169. 17984318 PMC2064788

[59] Vo T, Wang S, Poon GMK et al. Electrostatic control of DNA intersegmental translocation by the ETS transcription factor ETV6. J Biol Chem. 2017; 292:13187–96. 10.1074/jbc.M117.792887. 28592487 PMC5555182

[60] Li J, Rodriguez JP, Niu F et al. Structural basis for DNA recognition by STAT6. Proc Natl Acad Sci USA. 2016; 113:13015–20. 10.1073/pnas.1611228113. 27803324 PMC5135355

[61] Schulte KW, Green E, Wilz A et al. Structural basis for Aryl hydrocarbon receptor-mediated gene activation. Structure. 2017; 25:1025–33. 10.1016/j.str.2017.05.008. 28602820

[62] Wu D, Potluri N, Lu J et al. Structural integration in hypoxia-inducible factors. Nature. 2015; 524:303–8. 10.1038/nature14883. 26245371

[63] Srivastava J, Barber DL, Jacobson MP Intracellular pH sensors: design principles and functional significance. Physiology. 2007; 22:30–9. 10.1152/physiol.00035.2006. 17289928

[64] Liu Y, White KA, Barber DL Intracellular pH regulates cancer and stem cell behaviors: a protein dynamics perspective. Front Oncol. 2020; 10:1401 10.3389/fonc.2020.01401. 32983969 PMC7479815

[65] Nakagawa S, Gisselbrecht SS, Rogers JM et al. DNA-binding specificity changes in the evolution of forkhead transcription factors. Proc Natl Acad Sci USA. 2013; 110:12349–54. 10.1073/pnas.1310430110. 23836653 PMC3725104

[66] Forbes SA, Beare D, Boutselakis H et al. COSMIC: somatic cancer genetics at high-resolution. Nucleic Acids Res. 2017; 45:D777–83. 10.1093/nar/gkw1121. 27899578 PMC5210583

[67] Newman JA, Aitkenhead H, Gavard AE et al. The crystal structure of human forkhead box N1 in complex with DNA reveals the structural basis for forkhead box family specificity. J Biol Chem. 2020; 295:2948–58. 10.1074/jbc.RA119.010365. 31914405 PMC7062188

[68] Kim K, Lee S, Kang H et al. Dual specificity kinase dyrk3 promotes aggressiveness of glioblastoma by altering mitochondrial morphology and function. Int J Mol Sci. 2021; 22:2982 10.3390/ijms22062982. 33804169 PMC8000785

[69] Ponnusamy L, Natarajan SR, Manoharan R MARK2 potentiate aerobic glycolysis-mediated cell growth in breast cancer through regulating mTOR/HIF-1α and p53 pathways. J Cell Biochem. 2022; 123:759–71. 10.1002/jcb.30219. 35048405

[70] Ramella M, Ribolla LM, Surini S et al. Dual specificity kinase DYRK3 regulates cell migration by influencing the stability of protrusions. iScience. 2024; 27:109440 10.1016/j.isci.2024.109440. 38510137 PMC10952033

[71] Liesa M, Qiu W, Shirihai OS Mitochondrial ABC transporters function: the role of ABCB10 (ABC-me) as a novel player in cellular handling of reactive oxygen species. Biochim Biophys Acta Mol Cell Res. 2012; 1823:1945–57. 10.1016/j.bbamcr.2012.07.013. PMC3808996 22884976

[72] Diamant I, Clarke DJB, Evangelista JE et al. Harmonizome 3.0: integrated knowledge about genes and proteins from diverse multi-omics resources. Nucleic Acids Res. 2025; 53:D1016–28. 10.1093/nar/gkae1080. 39565209 PMC11701526

[73] Li G, Ma D, Chen Y Cellular functions of programmed cell death 5. Biochim Biophys Acta Mol Cell Res. 2016; 1863:572–80. 10.1016/j.bbamcr.2015.12.021. 26775586

[74] Li P, Fei H, Wang L et al. Pdcd5 regulates cell proliferation, cell cycle progression and apoptosis. Oncol Lett. 2018; 15:1177–83. 29403562 10.3892/ol.2017.7401 PMC5780840

[75] Park SY, Seo J, Choi HK et al. Protein serine/threonine phosphatase PPEF-1 suppresses genotoxic stress response via dephosphorylation of PDCD5. Sci Rep. 2017; 7:39222 10.1038/srep39222. 28051100 PMC5209732

[76] Arnold JM, Gu F, Ambati CR et al. UDP-glucose 6-dehydrogenase regulates hyaluronic acid production and promotes breast cancer progression. Oncogene. 2020; 39:3089–101. 10.1038/s41388-019-0885-4. 31308490 PMC6960374

[77] Morita T, Hayashi K Tumor progression is mediated by Thymosin-b4 through a TGFb/MRTF signaling axis. Mol Cancer Res. 2018; 16:880–93. 10.1158/1541-7786.MCR-17-0715. 29330296

[78] Makowiecka A, Malek N, Mazurkiewicz E et al. Thymosin β4 regulates focal adhesion formation in Human melanoma cells and affects their migration and invasion. Front Cell Dev Biol. 2019; 7:304 10.3389/fcell.2019.00304. 31921836 PMC6935720

[79] Kuo CY, Jhuang JY, Huang WC et al. Aberrant expression of thymosin beta-4 correlates with advanced disease and BRAF V600E mutation in thyroid cancer. J Histochem Cytochem. 2022; 70:707–16. 10.1369/00221554221138370. 36321670 PMC9660367

[80] Chi LH, Chang WM, Chang YC et al. Global proteomics-based identification and validation of thymosin beta-4 X-linked as a prognostic marker for head and neck squamous cell carcinoma. Sci Rep. 2017; 7:9031 10.1038/s41598-017-09539-w. 28831179 PMC5567379

[81] Bendris N, Schmid SL Endocytosis, metastasis and beyond: multiple facets of SNX9. Trends Cell Biol. 2017; 27:189–200. 10.1016/j.tcb.2016.11.001. 27989654 PMC5318277

[82] Bendris N, Williams KC, Reis CR et al. SNX9 promotes metastasis by enhancing cancer cell invasion via differential regulation of RhoGTPases. MBoC. 2016; 27:1409–19. 10.1091/mbc.E16-02-0101. 26960793 PMC4850029

[83] Karami-Tehrani F, Moeinifard M, Aghaei M et al. Evaluation of PDE5 and PDE9 expression in benign and malignant breast tumors. Arch Med Res. 2012; 43:470–5. 10.1016/j.arcmed.2012.08.006. 22960860

[84] Peng T, Gong J, Jin Y et al. Inhibitors of phosphodiesterase as cancer therapeutics. Eur J Med Chem. 2018; 150:742–56. 10.1016/j.ejmech.2018.03.046. 29574203

[85] Susmi TF, Rahman A, Khan MMR et al. Prognostic and clinicopathological insights of phosphodiesterase 9A gene as novel biomarker in human colorectal cancer. BMC Cancer. 2021; 21:577 10.1186/s12885-021-08332-3. 34016083 PMC8136133

[86] Fico F, Santamaria-Martínez A TGFBI modulates tumour hypoxia and promotes breast cancer metastasis. Mol Oncol. 2020; 14:3198–210. 10.1002/1878-0261.12828. 33080107 PMC7718944

[87] Corona A, Blobe GC The role of the extracellular matrix protein TGFBI in cancer. Cell Signal. 2021; 84:110028 10.1016/j.cellsig.2021.110028. 33940163

[88] Price MJ, Nguyen AD, Byemerwa JK et al. UDP-glucose dehydrogenase (UGDH) in clinical oncology and cancer biology. Oncotarget. 2023; 14:843–57. 10.18632/oncotarget.28514. 37769033 PMC10538703

[89] Amith SR, Wilkinson JM, Fliegel L Na+/H+ exchanger NHE1 regulation modulates metastatic potential and epithelial-mesenchymal transition of triple-negative breast cancer cells. Oncotarget. 2016; 7:21091–113. 10.18632/oncotarget.8520. 27049728 PMC5008271

[90] Siveen KS, Nizamuddin PB, Uddin S et al. TRPV2: a cancer biomarker and potential therapeutic target. Dis Markers. 2020; 2020:8892312 10.1155/2020/8892312. 33376561 PMC7746447

[91] Elbaz M, Ahirwar D, Xiaoli Z et al. TRPV2 is a novel biomarker and therapeutic target in triple negative breast cancer. Oncotarget. 2018; 9:33459–70. 10.18632/oncotarget.9663. 30323891 PMC6173360

[92] Samuelson AV, Narita M, Chan HM et al. p400 is required for E1A to promote apoptosis. J Biol Chem. 2005; 280:21915–23. 10.1074/jbc.M414564200. 15741165

[93] Mattera L, Escaffit F, Pillaire MJ et al. The p400/Tip60 ratio is critical for colorectal cancer cell proliferation through DNA damage response pathways. Oncogene. 2009; 28:1506–17. 10.1038/onc.2008.499. 19169279

[94] Macher-Goeppinger S, Bermejo JL, Schirmacher P et al. Senescence-associated protein p400 is a prognostic marker in renal cell carcinoma. Oncol Rep. 2013; 30:2245–53. 10.3892/or.2013.2698. 23982490

[95] Mao W, Guo Z, Dai Y et al. LNC CRYBG3 inhibits tumor growth by inducing M phase arrest. J Cancer. 2019; 10:2764–70. 10.7150/jca.31703. 31258784 PMC6584918

[96] Zheng L, Luo C, Yang N et al. Ionizing radiation-induced long noncoding RNA CRYBG3 regulates YAP/TAZ through mechanotransduction. Cell Death Dis. 2022; 13:209 10.1038/s41419-022-04650-x. 35246511 PMC8897501

[97] Soussi M, Hasselsweiller A, Gkika D TRP channels: the neglected culprits in breast cancer chemotherapy resistance?. Membranes. 2023; 13:788 10.3390/membranes13090788. 37755210 PMC10536409

[98] Lambert SA, Jolma A, Campitelli LF et al. The human transcription factors. Cell. 2018; 172:650–65. 10.1016/j.cell.2018.01.029. 29425488 PMC12908702

[99] Tencer AH, Gatchalian J, Klein BJ et al. A unique pH-dependent recognition of methylated histone H3K4 by PPS and DIDO. Structure. 2017; 25:1530–39. 10.1016/j.str.2017.08.009. 28919441 PMC5679713

[100] McBrian MA, Behbahan IS, Ferrari R et al. Histone acetylation regulates intracellular pH. Mol Cell. 2013; 49:310–21. 10.1016/j.molcel.2012.10.025. 23201122 PMC3893119

[101] Blane A, Fanucchi S Effect of pH on the structure and DNA binding of the FOXP2 forkhead domain. Biochemistry. 2015; 54:4001–7. 10.1021/acs.biochem.5b00155. 26055196

[102] Medina E, Villalobos P, Coñuecar R et al. The protonation state of an evolutionarily conserved histidine modulates domainswapping stability of FoxP1. Sci Rep. 2019; 9:5441 10.1038/s41598-019-41819-5. 30931977 PMC6443806

[103] Grillo-Hill BK, Choi C, Jimenez-Vidal M et al. Increased H+ efflux is sufficient to induce dysplasia and necessary for viability with oncogene expression. eLife. 2015; 4:e03270 10.7554/eLife.03270. 25793441 PMC4392478

[104] Kirsanov DD, Zanegina ON, Aksianov EA et al. NPIDB: nucleic acid–protein interaction database. Nucleic Acids Res. 2013; 41:D517–23. 10.1093/nar/gks1199. 23193292 PMC3531207

