# Supplementary material for: A role for pH dynamics regulating transcription factor DNA-binding selectivity
Source: Nucleic Acids Res. 2025 Jun 4;53(10):gkaf474. doi: 10.1093/nar/gkaf474 (PMC12135187; doi:10.1093/nar/gkaf474)
Supplement: gkaf474_Supplemental_Files [file gkaf474_supplemental_files.zip › Supplementary Data_Reformat_Final_040925.pdf]

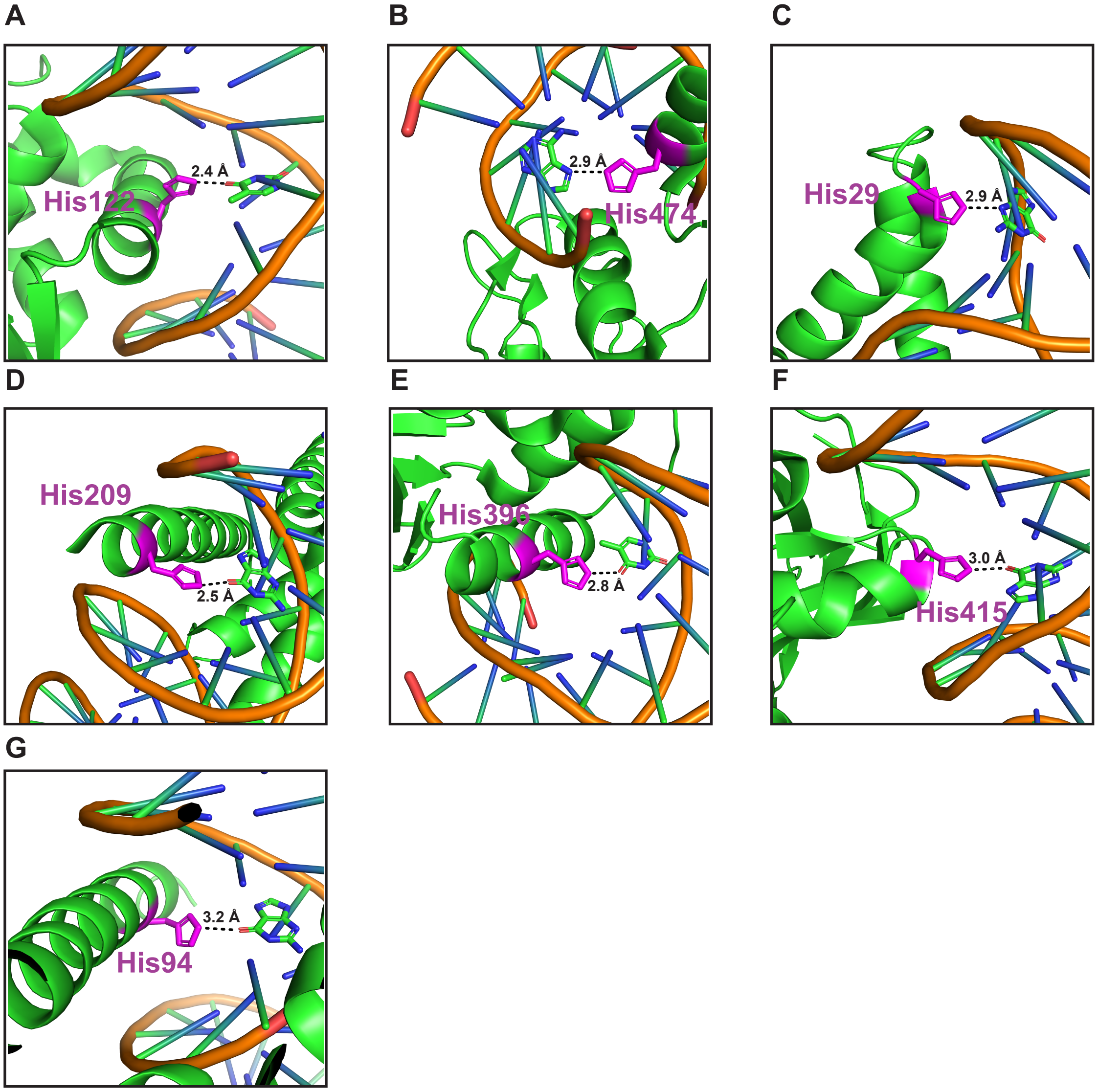
A
2.4 Å
His122
B
2.9 Å
His474
C
His29
2.9 Å
D
His209
2.5 Å
E
His396
2.8 Å
F
3.0 Å
His415
G
3.2 Å
His94

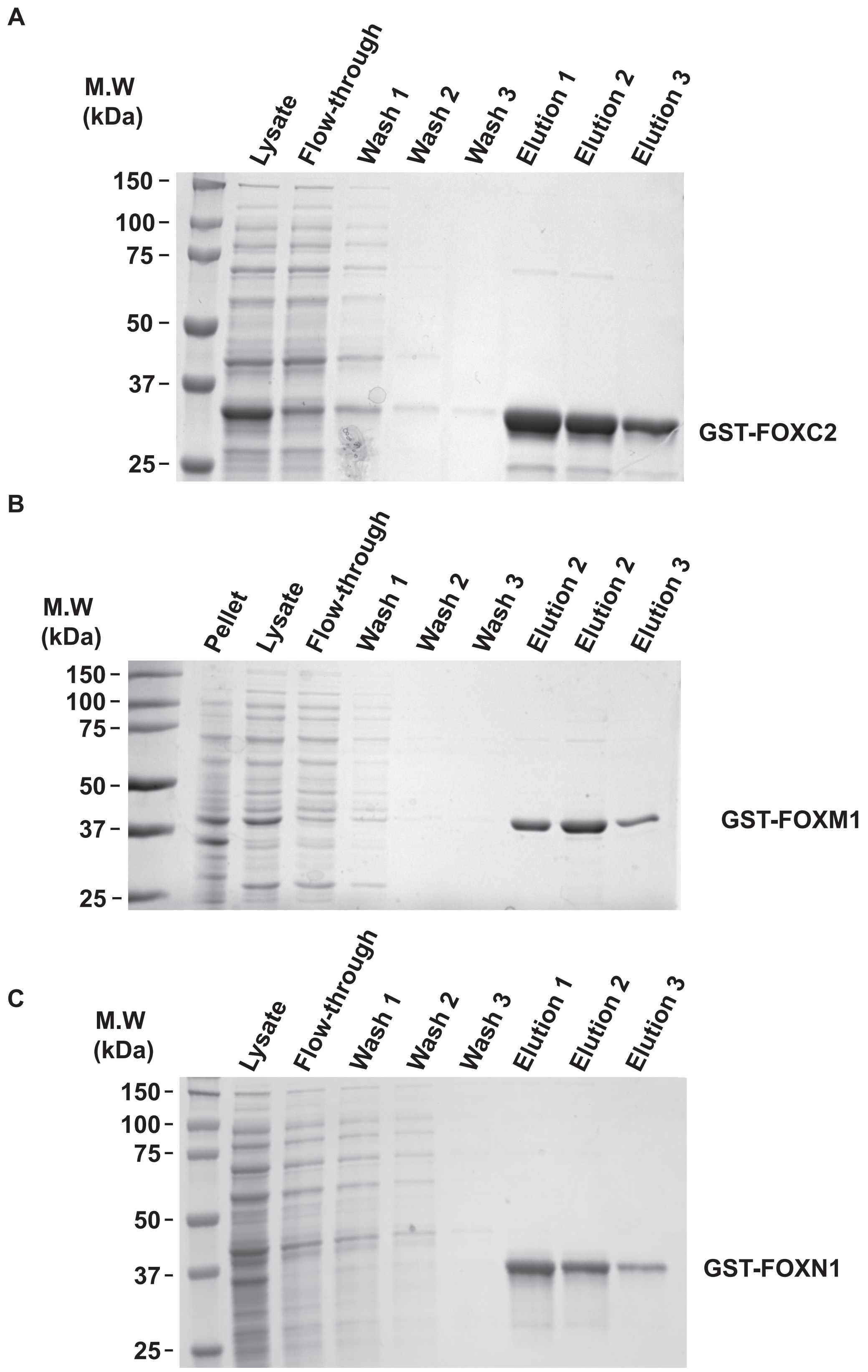
A
M.W (kDa)
Lysate
Flow-through
Wash 1
Wash 2
Wash 3
Elution 1
Elution 2
Elution 3
150
100
75
50
37
25
GST-FOXC2
B
M.W (kDa)
Pellet
Lysate
Flow-through
Wash 1
Wash 2
Wash 3
Elution 2
Elution 2
Elution 3
150
100
75
50
37
25
GST-FOXM1
C
M.W (kDa)
Lysate
Flow-through
Wash 1
Wash 2
Wash 3
Elution 1
Elution 2
Elution 3
150
100
75
50
37
25
GST-FOXN1

A

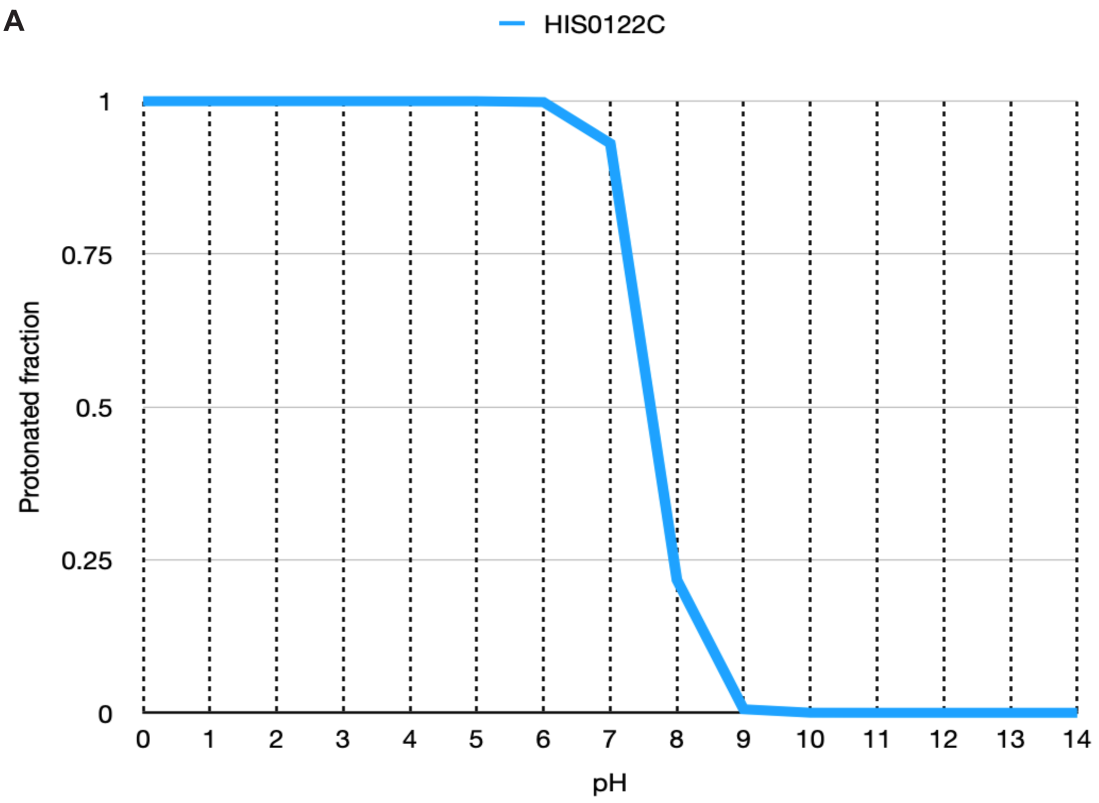
HIS0122C
Protonated fraction
1
0.75
0.5
0.25
0
0
1
2
3
4
5
6
7
8
9
10
11
12
13
14
pH

B

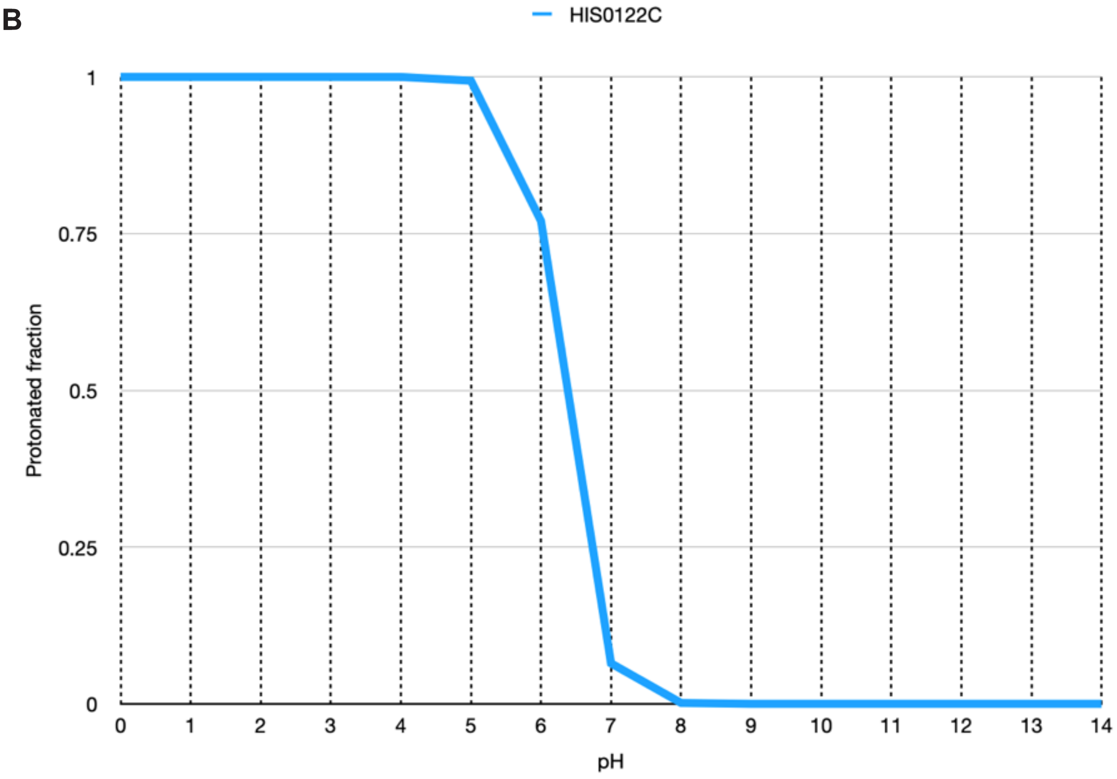
HIS0122C
Protonated fraction
1
0.75
0.5
0.25
0
0
1
2
3
4
5
6
7
8
9
10
11
12
13
14
pH

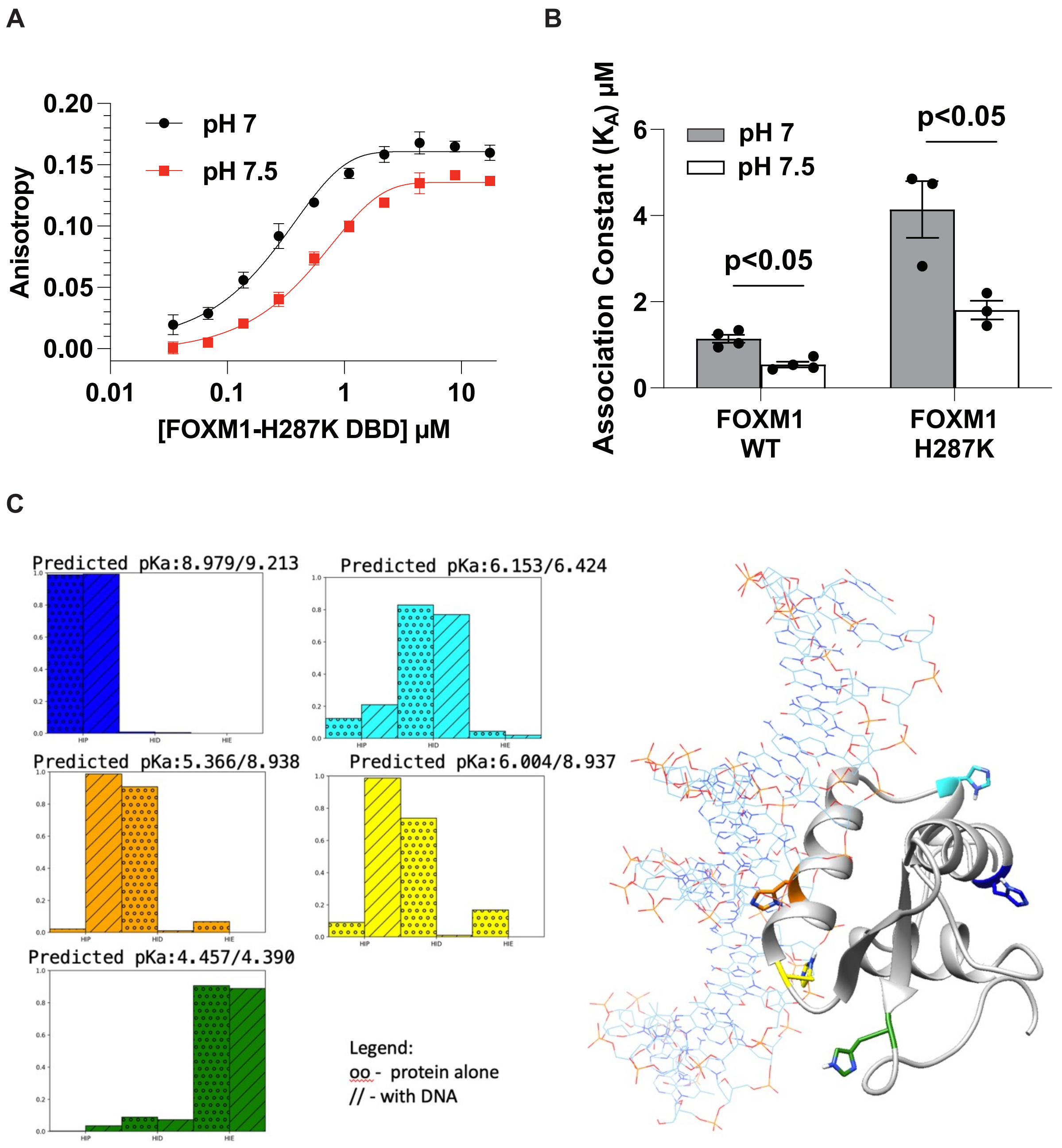
A
Anisotropy
0.20
0.15
0.10
0.05
0.00
pH 7
pH 7.5
0.01
0.1
1
10
[FOXM1-H287K DBD] µM
B
Association Constant (KA) µM
6
4
2
0
pH 7
pH 7.5
p<0.05
p<0.05
FOXM1 WT
FOXM1 H287K
C
Predicted pKa:8.979/9.213
Predicted pKa:6.153/6.424
Predicted pKa:5.366/8.938
Predicted pKa:6.004/8.937
Predicted pKa:4.457/4.390
Legend:
oo - protein alone
// - with DNA

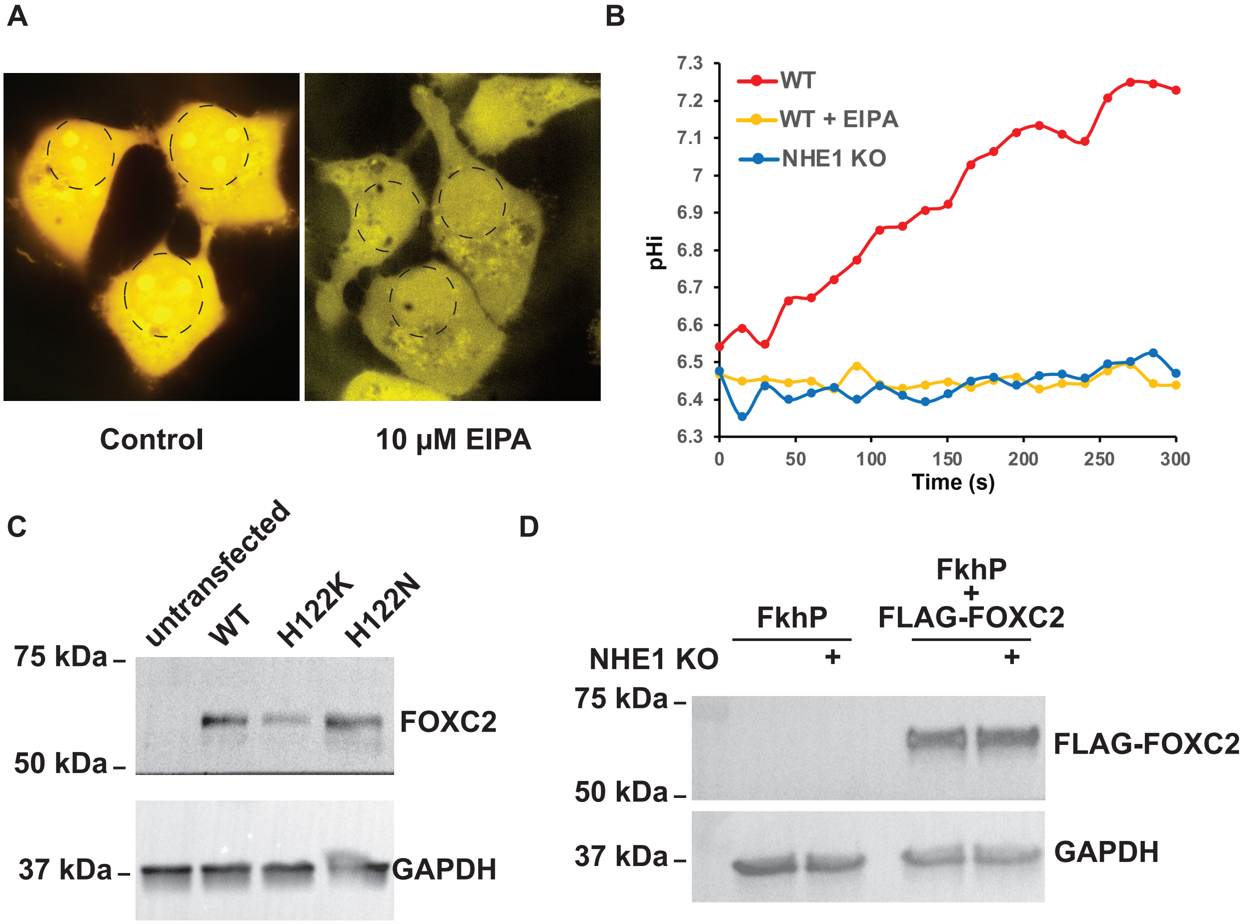
A
B
Control
10 μM EIPA
WT
WT + EIPA
NHE1 KO
pHi
Time (s)
7.3
7.2
7.1
7
6.9
6.8
6.7
6.6
6.5
6.4
6.3
0
50
100
150
200
250
300
C
untransfected
WT
H122K
H122N
75 kDa
50 kDa
37 kDa
FOXC2
GAPDH
D
FkhP
FkhP + FLAG-FOXC2
NHE1 KO
+
+
75 kDa
50 kDa
37 kDa
FLAG-FOXC2
GAPDH

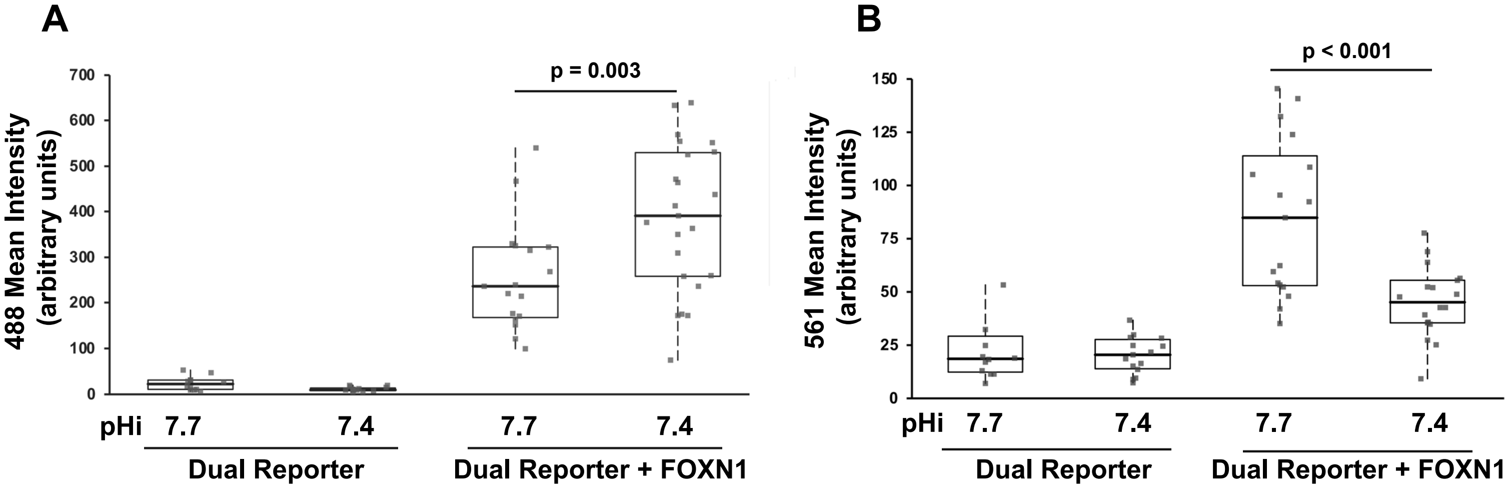
A
p = 0.003
488 Mean Intensity
(arbitrary units)
700
600
500
400
300
200
100
0
pHi
7.7
7.4
7.7
7.4
Dual Reporter
Dual Reporter + FOXN1
B
p < 0.001
561 Mean Intensity
(arbitrary units)
150
125
100
75
50
25
0
pHi
7.7
7.4
7.7
7.4
Dual Reporter
Dual Reporter + FOXN1

Supplemental Figure Legends

Supplementary Figure S1. Hydrogen bonding by conserved histidine in the DBD of transcription factors in different families.

A-D. Representative transcription factors from families with conserved histidine (magenta) forming a hydrogen bond with DNA nucleotides, including FOXC2-His122 binding to thymine (PDB:6AKO) (A), KLF4-His474 binding to adenine (PDB: 2WBU) (B), SOX4-His29 binding to guanine (PDB: 3U2B) (C), and MITF-His209 binding to guanine (PDB: 4ATK) (D). E-G. Select transcription factors in other families shown to have a hydrogen bond between histidine (magenta) and a DNA nucleotide, including ETV6-His396 binding to thymine (PDB: 4MHG) (E), STAT6-H415 binding to guanine (PDB:4Y5W) (F), and ARNT-His94 binding to guanine (PDB: 4ZPK) (G).

Supplementary Figure S2. Purified GST fusion proteins of FOXC2, FOXM1, and FOXN1 DBD.

A-C. Coomasie stained gels of purified GST fusions of the DBDs of FOXC2 (A), FOXM1 (B), and FOXN1 (C).

Supplementary Figure S3. **DelPhiPKa predicted titration curve of DNA bound and unbound His122.**

A. FOXC2 DNA-bound His122 titration curve generated with DelPhiPKa webserver. His122 predicted pKa: 7.75. B FOXC2 unbound His122 titration curve generated with DelPhiPKa webserver. His122 predicted pKa: 6.24.

Supplementary Figure S4. Measured and predicted binding of FOXM1-H287K to an FkhP sequence.

A, B. Binding of recombinant DBD of FOXM1-H287K, including binding curves at the indicated pH values, determined by fluorescence anisotropy (A) and association constants calculated from binding curves (B). Data are means ± s.e.m. of three separate measurements with two independent protein preparations. C. CpHMD portion of simulation in which FOXM1 DBD HIS residues are double protonated (HIP), protonated at delta nitrogen (HID), protonated at epsilon nitrogen (HIE) are predicted to be unbound (circles) or bound FkhP (slashes). All DBD HIS residues are labeled His269 (blue), His275 (cyan), His287 (orange), His292 (yellow), His311 (green).

Supplementary Figure S5. Loss of NHE1 activity and expression of recombinant FOXC2 in NHE1-KO MDA-MB-436 cells.

A. Confocal images of control and EIPA treated MDA-MB-436 cells loaded with SNARF. Nuclei are indicated by dashed circles. B. The pHi recovery in a nominally-HCO_3-free Hepes buffer from an NH_4Cl-induced acid load, an index of NHE1-dependent H+ extrusion, in the indicated MDA-MB-436 cells in the absence and presence of EIPA and with NHE1-KO loaded with the pH-sensitive dye BCECF. C. Expression of endogenous FOXC2 compared with transiently transfected WT or pH-independent mutants. D. FOXC2 expression from luciferase assay lysates in controls and NHE1-KO MDA-MB-436 cells.

Supplementary Figure S6. Fluorescence images with dual reporter and mean intensity of mKate2 and eGFP.

A,B. Mean intensity of dual reporter alone or dual reporter co-expressed with FOXN1 for eGFP (Ex488; A) and for mKate2 (Ex561; B). Box plots show median, first and third quartile, with whiskers extending to observations within 1.5 times the interquartile range, and individual data points representing values from a single image with at least five images analyzed in three separate cell preparations. Statistical analysis by Tukey-Kramer HSD test.

Supplemental File 1

SELEX-seq results with DNA motif (Kmer) and corresponding enrichment score.

Supplemental File 2

FOXC2 ChIP-seq results with annotated peaks for control and NHE1-KO MDA-MB-436 cells.

Supplemental File 3

RNA-seq results for untransfected control compared to NHE1-KO MDA-MB-436 cells.

Supplemental File 4

RNA-seq results for FOXC2-WT transfected control compared to NHE1-KO MDA-MB-436 cells.

Supplemental File 5

RNA-seq results for FOXC2-H122K transfected control compared to NHE1-KO MDA-MB-436 cells.

Supplemental File 6

RNA-seq results for FOXC2-H122N transfected control compared to NHE1-KO MDA-MB-436 cells.
